# Open- and Closed-Shell Roles of Sensitizer and Annihilator in Pseudo-Single Component Mixtures for Upconversion

**DOI:** 10.1021/jacs.6c04090

**Published:** 2026-07-03

**Authors:** Kieran D. Richards, Wenzhao Wang, Philipp Thielert, James D. Green, John M. Hudson, Claire Tonnelé, David Casanova, Yoann Olivier, Timothy J. H. Hele, Sabine Richert, Feng Li, Emrys W. Evans

**Affiliations:** † Department of Chemistry, 7759 Swansea University, Singleton Park, Swansea SA2 8PP, United Kingdom; ‡ Centre for Integrative Semiconductor Materials, Swansea University, Fabian Way SA1 8EN, United Kingdom; § State Key Laboratory of Supramolecular Structure and Materials, College of Chemistry, 12510 Jilin University, Qianjin Avenue 2699, Changchun 130012, P. R. China; ∥ Institute of Physical Chemistry, University of Freiburg, Albertstraße 21, 79104 Freiburg, Germany; ⊥ Department of Chemistry, University College London, WC1H 0AJ London, U.K.; # 226245 Donostia International Physics Centre, 20018 Donostia, Euskadi, Spain; ∇ Ikerbasque, Basque Foundation for Science, 48009 Bilbao, Euskadi, Spain; ○ Laboratory for Computational Modelling of Functional Materials, Namur Institute of Structured Matter, University of Namur, Rue de Bruxelles 61, 5000 Namur, Belgium

## Abstract

Photochemical upconversion by annihilation of two triplet excitons to a higher-energy singlet state enables energy control of photons in optoelectronics and photonics. Upconversion initiated by a closed-shell sensitizer is limited by energy losses from singlet–triplet intersystem crossing. Here we explore open-shell organic radicals as sensitizers and their closed-shell hydrogenated analogues as annihilators in photon upconversion. The sensitizer combines optical transitions from a triphenylmethyl (TTM-1Cz) radical with energy-degenerate triplet states of an anthracene-based component (DPA) in one molecule (TTM-1Cz-DPA). The difference of one hydrogen atom in its closed-shell counterpart (HTTM-1Cz-DPA) switches the spin-optical properties to an annihilator that can mediate efficient upconversion. Red-to-blue photon upconversion by intermolecular energy transfer, from open-shell sensitizer to closed-shell annihilator, is demonstrated in solution with an apparent anti-Stokes shift higher than 0.9 eV and 7% quantum efficiency. We evaluate this mechanism against true ‘single component’ mixtures for upconversion and find that the presence of hydrogenated precursor with radicals is essential for high performance. Understanding the emergent spin-optical properties from paired open-shell and closed-shell systems enables new opportunities for energy management from the same molecular frame.

## Graphical abstract

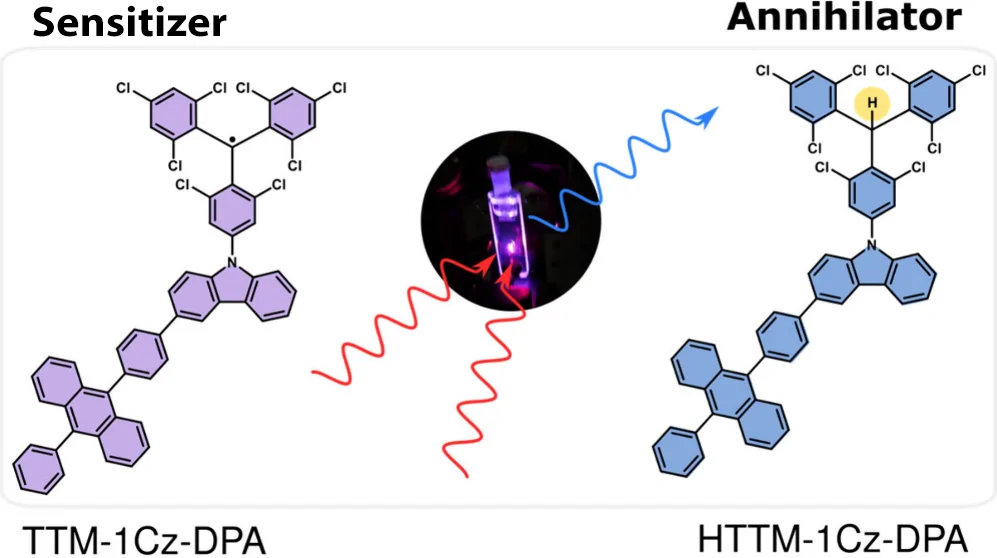

## 1 Introduction

Molecular photochemical upconversion by triplet–triplet annihilation (TTA) enables the basis for current and future technologies in bioimaging, photodynamic therapy, renewable energy production and photocatalysis.^1–3^ The transformation of low to high energy photons is typically realized by two-component molecular systems. A sensitizer molecule acts as a light absorber that forms triplet (T_1_) excitons by intersystem crossing (ISC) from singlet (S_1_) states following photoexcitation. Sensitizers based on organometallic compounds with enhanced ISC from strong spin–orbit coupling have been employed to generate long-lived triplet states.^4–12^ Triplet states are initiated on a second annihilator molecule by energy transfer from the sensitizer to the annihilator.^13^ Subsequent annihilation of triplet excitons leads to P-type delayed fluorescence from a singlet state that emits higher energy photons (*hv’*) compared to those initially absorbed by the sensitizer (*hv*): T_1_ + T_1_ → S_1_ + S_0_ → 2S_0_ + *hv’*. Established TTA molecules based on anthracene such as 9,10-diphenylanthracene (DPA) and 9,10-bis (phenylethynyl)­anthracene (BPEA) have high photoluminescence quantum efficiency (PLQE) (up to ∼100%) and high annihilation rate constants (>10^9^ M^–1^ s^–1^) that maximize the upconversion emission efficiency.^14^ The indirect mechanism to initiate T_1_ states has the disadvantage of reducing the maximum upconversion energy shift due to losses from the singlet–triplet energy gap (Δ*E*_ST_) of the sensitizer. Larger upconversion shifts were explored in various designs of closed-shell sensitizers including thermally activated delayed fluorescence (TADF) molecules with reduced Δ*E*_ST_ and those with enhanced optical transitions from the ground state to T_1_.^15–19^ However, achieving a high triplet density by these approaches is challenging because of an inherently low ISC rate, limited by weak spin orbit coupling in TADF molecules and weak molar absorption coefficients for S_0_ → T_1_ absorption.

Radical-enhanced ISC following photoexcitation can lead to efficient T_1_ formation in organic chromophores coupled to nonconjugated radicals, such as (2,2,6,6-tetramethylpiperidin-1-yl)­oxyl (TEMPO), as alternative sensitizers for upconversion.^20^ In this design, the maximum upconversion energy shift is still limited by Δ*E*_ST_ of the chromophore moiety.^21,22^

Photochemical upconversion was demonstrated with tris­(2,4,6-trichlorophenyl)­methyl (TTM)-based radicals acting as the chromophore and sensitizer components (Figure 1a).^23,24^ The radical moiety can be substituted by carbazole (TTM-1Cz) and triphenylamine (TTM-TPA) moieties to control the energy of their photoexcited D_1_ states. In these systems, near resonant intermolecular doublet–triplet energy transfer toward the annihilator T_1_ state can initiate upconversion without substantial energy loss. However, the relatively short D_1_ lifetime (<50 ns) of these organic radical materials in toluene solution leads to moderate intermolecular doublet–triplet energy transfer efficiency. For TTM-1Cz/annihilator where [annihilator] = 10 mM, the energy transfer efficiency, Φ_IET_, is ∼20% for quenching rate constant, *k*_q_ = 10^9^ M^–1^s^–1^, and Φ_IET_ ∼ 3% if *k*_q_ = 10^8^ M^–1^ s^–1^.^23^ For an intermolecular system with TTM-1Cz as sensitizer and DPA as annihilator, the upconversion quantum efficiency is Φ_UC_ = 0.13%.^23^ The short excited-state lifetime of π-radical sensitizers imposes a fundamental limit on upconversion performance that must be improved for future applications.

**Figure 1:**
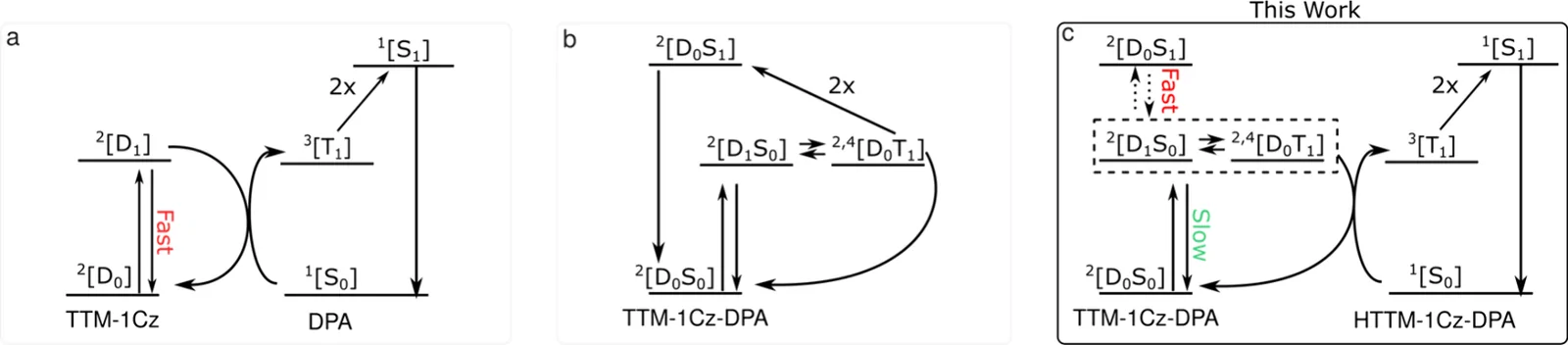
Energy level diagrams of photochemical upconversion for sensitizer:annihilator systems using (a) radical sensitizer; (b) radical-annihilator dyad which acts as sensitizer and annihilator; and (c) radical-annihilator dyad as a sensitizer with its closed-shell analogue as an annihilator, which differs by a single hydrogen atom.

The concept of extended lifetimes for triplet sensitization was previously demonstrated by closed-shell sensitizer–annihilator dyads based on organometallic (Ru, Ir, Os) and thermally activated delayed fluorescence sensitizers.^25–30^ This was translated to open-shell Fe-based systems exploiting doublet states, in which ∼100 ns excited state lifetime for the sensitizer was realized.^31^

Recently we explored TTM-1Cz radicals coupled to anthracene in luminescent radical–triplet dyads as molecular quantum systems that enable initiation and read-out of high-spin multiplicity states (*S* > 1) with light.^32^ The spin-optical interface relies on reversible intramolecular energy transfer between the D_1_ state of TTM-1Cz to a trip-doublet ^2^[D_0_T_1_] and trip-quartet ^4^[D_0_T_1_] manifold where a local triplet excitation on anthracene is coupled to the radical in overall doublet and quartet states. This enables an extended excited-state lifetime of radicals (>50 ns) beyond their radiative limit, which can also benefit more intermolecular triplet sensitization in photochemical upconversion.

An investigation of radical-annihilator dyads using covalently linked TTM-1Cz (radical) and perylene (annihilator) has proposed the single component concept of sensitizer and annihilator action by the same molecule with radicals (Figure 1b).^33^ First, as a sensitizer, photoexcitation of the radical moiety at 610 nm results in intramolecular energy transfer to the perylene T_1_ state and an extended radical-annihilator excited state (97 ns) for sensitization. Second, as an annihilator, the study proposed that annihilation of two perylene T_1_ excitations between two TTM-1Cz-perylene radicals yields upconverted photoluminescence at 475 nm from ^2^[D_0_T_1_]. An upconversion quantum efficiency of Φ_UC_ = 0.012% was reported.^33^ However, the proposed mechanism may be unviable if upconverted emission is outcompeted by internal conversion from ^2^[D_0_S_1_] → ^2^[D_1_S_0_], as would be expected from Kasha’s Rule. Alternative mechanisms to understand and exploit photochemical upconversion from radical-annihilator dyads should be considered.

Here we explore the exchange from sensitizer to annihilator by open-shell radicals and their closed-shell counterparts based on the same molecular frame in photochemical upconversion. TTM-1Cz-DPA is our prototype open-shell radical sensitizer dyad in which TTM-1Cz is covalently linked to the benchmark DPA annihilator (Figure 2a). The synthesis of TTM-1Cz-DPA was previously reported, but its spin-optical properties and application in photochemical upconversion were not investigated.^33^

**Figure 2:**
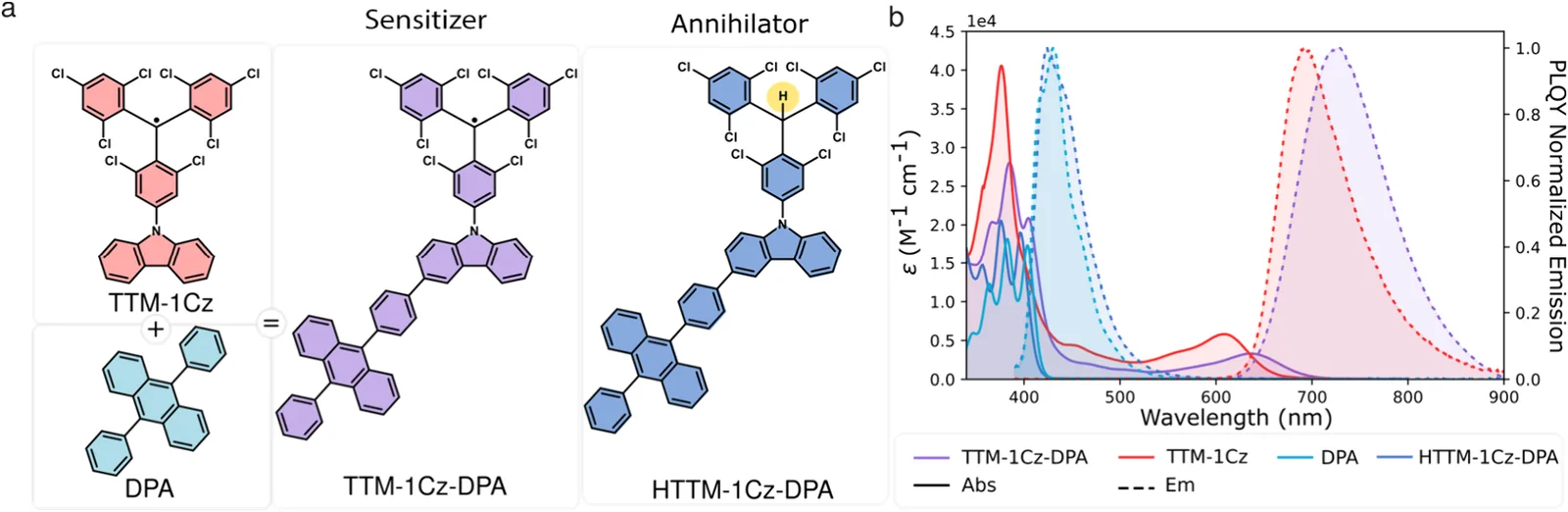
(a) Chemical structures of open-shell TTM-1Cz, DPA (9,10-diphenylanthracene), open-shell TTM-1Cz-DPA and closed-shell HTTM-1Cz-DPA. (b) Absorption and photoluminescence spectra of the molecules in toluene.

Following photoexcitation of the radical moiety in TTM-1Cz-DPA, we find that triplets of DPA are formed within the trip-doublet and trip-quartet states of the dyad by performing transient optical and electron spin resonance studies. This results in an extended excited-state lifetime (>250 ns) of TTM-1Cz-DPA compared to the TTM-1Cz radical component (Figure 1c). The substitution from ‘C•’ to ‘C–H’ in the TTM moiety from TTM-1Cz-DPA to HTTM-1Cz-DPA switches the spin-optical manifold to singlet–triplet photophysics derived from the DPA group. We demonstrate that efficient red-to-blue light upconversion requires intermolecular energy transfer and can be mediated by open-shell TTM-1Cz-DPA sensitizer and closed-shell HTTM-1Cz-DPA annihilator components in solution. An apparent anti-Stokes energy shift of 0.9 eV is observed with 7% and 12% upconversion quantum efficiency for TTM-1Cz-DPA paired with HTTM-1Cz-DPA and DPA, respectively. This demonstrates exceptional upconversion efficiency for all-organic radical materials. Simultaneously, no single component upconversion was found for the materials presented here, suggesting that not all annihilator-linked radicals exhibit anti-Kasha emission, or that hydrogenated impurities of radicals can play a role in upconversion. We hereby present the possibility of combining radicals and their precursor based on the same molecular frame as a ‘pseudo-single component’ system for upconversion. Our work advances the understanding and opportunities of energy and photon management from exploiting the photophysics of open-shell and closed-shell materials together.

## 2 Results and Discussion

### 2.1 Optical Properties of TTM-1Cz-DPA

The annihilator-coupled π-radical, TTM-1Cz-DPA (Figure 2a), was synthesized as described in the Supporting Information. In Figure 2b, the UV–vis absorption spectrum of TTM-1Cz-DPA reflects its constituent parts of TTM-1Cz and DPA. The peak absorption feature at 385 nm shows DPA character, corresponding to the ^2^[D_0_S_0_] → ^2^[D_0_S_1_] transition, and its vibronic progression combined with the TTM absorption signature.^34^ The peak at 635 nm reflects the radical TTM-1Cz component associated with the ^2^[D_0_S_0_] → ^2^[D_1_S_0_] transition.^23^ The steady-state photoluminescence (PL) of TTM-1Cz-DPA is centered at 725 nm in toluene and reflects TTM-1Cz-based emission. A PLQE of 5.0% was measured for TTM-1Cz-DPA in toluene. Both the absorption and the PL spectra appear red-shifted compared to their constituents, which can be attributed to the extended π-conjugation in TTM-1Cz-DPA.

The closed-shell analogue HTTM-1Cz-DPA shows UV–vis absorption and emission profiles that reflect the DPA component (Figure 2b). The lowest energy absorption peak at 396 nm corresponds to the 0,0 transition for ^1^[S_0_] → ^1^[S_1_], with a corresponding emission peak at 424 nm. A vibronic progression in absorption is observed with further peaks at 376, 357, and 322 nm. The PLQE was found to be 72% in toluene. As expected, quantum-chemical calculations of HTTM-1Cz-DPA show that the frontier orbitals are localized on the DPA moiety, and that the lower-lying singlet and triplet excited states are dominated by the HOMO to LUMO transition (SI Figure S6, Table S1). The substitution from ‘C•’ to ‘C–H’ in the TTM moiety establishes electronic and optical properties similar to DPA in HTTM-1Cz-DPA, as reflected in Figure 2b. This enables the possibility of radicals (TTM-1Cz-DPA) and their precursors (HTTM-1Cz-DPA) to act in sensitizer and annihilator roles for ‘pseudo-single’ component upconversion. For clarity, we highlight that true single component upconversion in this context would be systems containing the radical (TTM-1Cz-DPA) only.

### 2.2 Exciton Dynamics and Spin Properties of TTM-1Cz-DPA

Transient photophysics and electron spin resonance studies of TTM-1Cz-DPA were performed to investigate the exciton dynamics and spin properties of its excited-state manifold.

Femtosecond transient absorption studies of 50 μM TTM-1Cz-DPA in toluene (Figure 3a) show a picosecond decay of the TTM-1Cz doublet exciton absorption signatures (500–600 nm and 700–850 nm) and a reciprocal rise of the DPA triplet features (410–450 nm) after 635 nm laser excitation. Two time constants are identified by global analysis (9.4 ps and >7 ns, outside the experiment range), and the corresponding decay associated spectra (DAS) are given in Figure 3b. We attribute the 9.4 ps event to the internal conversion of ^2^[D_1_S_0_] → ^2^[D_0_T_1_], showing a negative DAS at 410–450 nm, where the formation of triplet excitons of the anthracene moiety from the excited radical group follows a spin-conserved intramolecular doublet energy transfer process.^32^ This is also supported by the observation that the ground state bleach region (380–410 nm) in the DAS for 9.4 ps and >7 ns decays reflect the absorption profiles of TTM-1Cz and DPA, respectively, including the latter’s vibronic progression.

**Figure 3:**
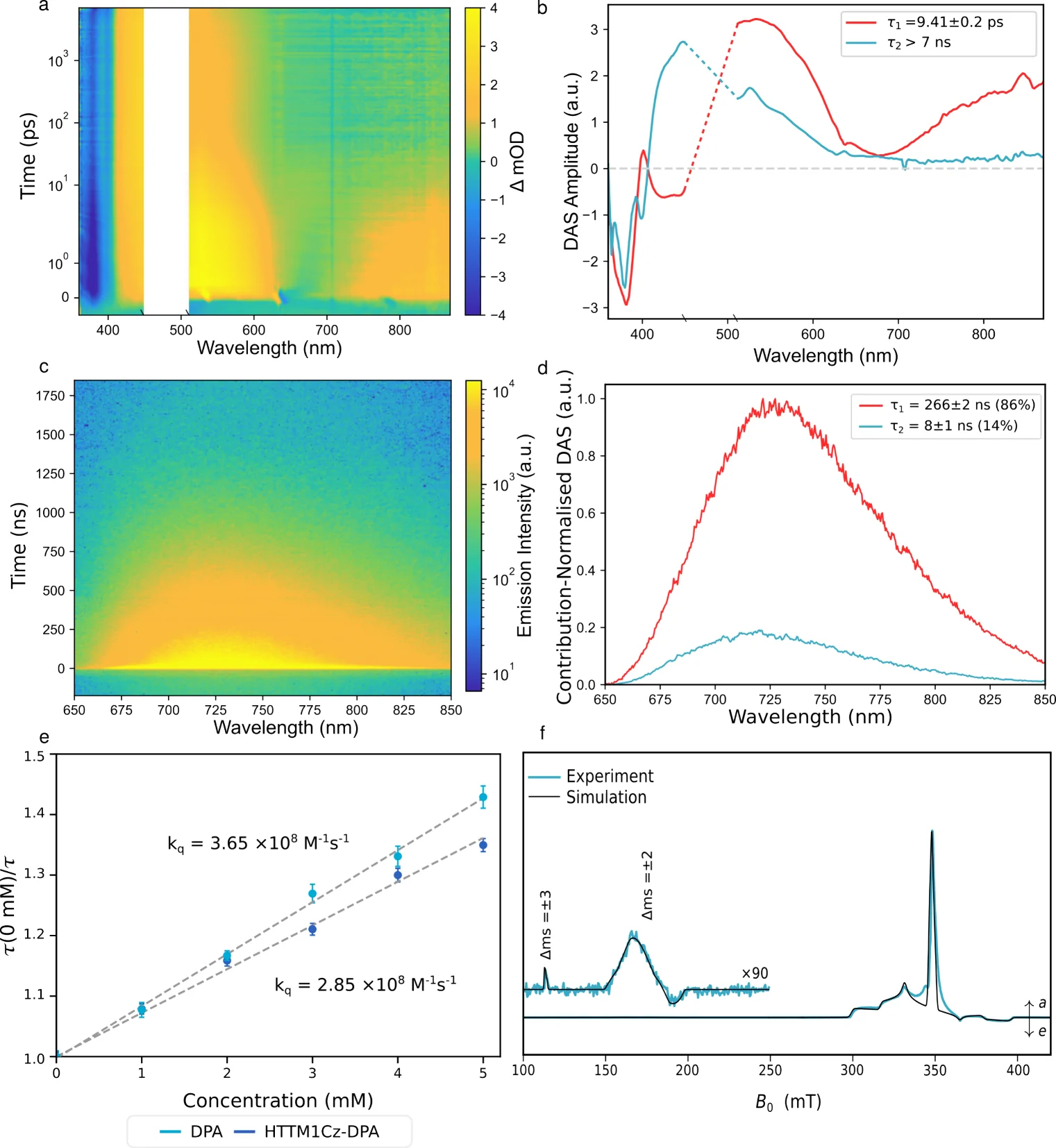
(a) Femtosecond transient absorption (TA) spectra of TTM-1Cz-DPA following 635 nm excitation (17 mJ cm^–2^). (b) TA Decay associated spectra obtained by global analysis, showing two time constants at <10 ps and >7 ns, longer than the instrument collection window. An axis break is present between UV and VIS probe regions – shown by dashed lines. (c) Transient PL (TrPL) of the emission from TTM-1Cz-DPA following 600 nm excitation (7 μJ cm^–2^). (d) TrPL Decay Associated Spectra (DAS) obtained by global analysis, showing two time constants at 8 and 266 ns. (e) Stern–Volmer plot for the PL quenching of TTM-1Cz-DPA by DPA and HTTM-1Cz-DPA, using TrPL (see Figures S17 and S18). All studies were performed at room temperature in toluene. (f) Transient continuous wave ESR spectrum of TTM-1Cz-DPA (200 μM) after laser excitation at 600 nm, recorded in frozen toluene solution at 80 K (blue) with the fitted simulation of a triplet–doublet pair (black). In addition to the main signal, the formally forbidden ESR transitions associated with Δm_S_ = ±2 and Δm_S_ = ±3 are observed (zoom inset).

The transient absorption spectra for TTM-1Cz-DPA excitation at 400 nm are given in the Supporting Information (Figure S3). The main difference observed is that the shorter time constant has increased from 9.4 to 14.7 ps. This may be attributed to contributions from consecutive internal conversion steps of ^2^[D_0_S_1_] → ^2^[D_1_S_0_] and then ^2^[D_1_S_0_] → ^2^[D_0_T_1_]. The ∼1 eV energy gap between ^2^[D_0_S_1_] and ^2^[D_1_S_0_] is bridged by radical-centered excited states (i.e., ^2^[D_*i*>1_S_0_]) as visible from (weak) absorption features between 385 and 635 nm in the UV–vis spectrum (Figure 2b). Our findings suggest that emission from the higher-lying ^2^[D_0_S_1_] state is outcompeted by relaxation, which precludes the annihilator action for TTM-1Cz-DPA. It is not clear how molecules can be designed to disobey Kasha’s Rule as previously claimed in single-component upconversion with radicals.^33^ We consider the role of TTM-1Cz-DPA as a sensitizer only in upconversion.

Figure 3c and d show the transient PL (TrPL) kinetics of TTM-1Cz-DPA from ns to μs time scales, monitored between 650 and 850 nm from sample excitation at 600 nm. The data display a prompt (8 ± 1 ns) and delayed (266 ± 2 ns) PL component from Decay Associated Spectra (DAS) that matches the steady-state profile in Figure 2b, which suggests the same molecular origin of light emission across time scales. We relate this to reversible transfer between the trip-doublet and trip-quartet states, ^2,4^[D_0_T_1_], and to the luminescent ^2^[D_1_S_0_] doublet state of the radical. The lifetime of the delayed emission is markedly longer than that of the parent TTM-1Cz radical (27 ns in toluene).^23^ The extended lifetime of these states is important to enable an improved sensitization efficiency by radicals when applied in photochemical upconversion.

Electron spin resonance (ESR) studies were conducted to characterize the spin properties of the ground and excited states in TTM-1Cz-DPA. Continuous wave ESR spectra of TTM-1Cz-DPA were recorded at the X-band (9.75 GHz) at room temperature and display a narrow signal centered at *g* = 2.00365 (see Figure S16), which is characteristic of TTM derivatives and corresponds to the radical ground state, D_0_ or ^2^[D_0_S_0_] in Figure 1c.

The transient continuous wave ESR spectrum of TTM-1Cz-DPA, following 600 nm excitation in frozen toluene (200 μM, 80 K), is shown in Figure 3f. The main signal (Δ*m*_S_ = 1) is characterized by a broad multiplet polarization with an approximate width of ∼100 mT and an intense absorptive net polarization. The overall spectral width is clearly reduced compared to the typical transient ESR spectrum of an anthracene triplet state with a width of ∼150 mT and the intense net polarization in the center of the spectrum is typical for a quartet state ( $\left|m_{S}=-1/2\right\rangle ↔\left|m_{S}=+1/2\right\rangle$ transition). The quartet nature of the signal is also confirmed by the observation of the forbidden Δ*m*_S_ = ± 2 and Δ*m*_S_ = ± 3 transitions at roughly half and one-third of the magnetic field, compared to the main signal. These transitions are much less intense, which is why this region is scaled by a factor of 90 in Figure 3f. The spectrum is simulated as a strongly coupled triplet–doublet pair and shows good agreement for the main signal and forbidden transitions. The details of the simulation and the simulation parameters can be found in the Supporting Information (Table S8). The transient ESR signals in half- and full-field regions can be modeled by doublet/quartet-spin exciton formation in ^2,4^[D_0_T_1_], similar to our previous work and others on radical–chromophore coupled systems.^22,32,35–41^ Overall the ESR studies support that ‘preformed’ triplets from the DPA moiety are generated within TTM-1Cz-DPA following photoexcitation.

### 2.3 Intermolecular Triplet Energy Transfer from TTM-1Cz-DPA

The spin and photophysics studies of TTM-1Cz-DPA support an excited-state manifold with ‘preformed’ triplet states ^2^[D_0_T_1_], ^4^[D_0_T_1_] and reversible transfer with long-lived, radical-based excitations ^2^[D_1_S_0_]. In order to understand the possible mechanisms for triplet sensitization from this manifold in Figure 1c, we consider the energy transfer from the underlying sensitizer excited states to form annihilator T_1_ from a theoretical and modeling perspective (see Supporting Information, Section 3).^32,34,42–48^

We performed quantum chemical calculations on both the sensitizer alone (TTM-1Cz-DPA) and dimers of the sensitizer and DPA annihilator. Time-dependent density functional theory (TD-DFT) calculations on TTM-1Cz-DPA revealed an electronic structure and intramolecular photophysics similar to the TTM-1Cz-anthracene compound previously characterized for spin-optical interface applications (see Supporting Information Section 33).^32^

Dispersion-corrected DFT calculations were conducted on the radical sensitizer:annihilator model system, TTM-1Cz-DPA:DPA. The calculations show that different stable arrangements of the sensitizer:annihilator are possible with the annihilator being close either to the radical or the DPA moiety of the sensitizer. The calculation of the excitonic coupling revealed that efficient energy transfer mechanisms to the annihilator can occur through either direct two-electron exchange or mediated via ^2^[intra-CT] states (Figure S15) and is considered further in the Supporting Information (‘Quantum Chemical Calculations’).

In order to develop our molecular understanding of the process, Hamiltonian interaction elements for intermolecular energy transfer were derived by using electronic structure theory. Our analysis finds that energy transfer by one-step Dexter and charge transfer mechanisms from ^2,4^[D_0_T_1_] and ^2^[D_1_S_0_] are all quantum mechanically allowed as additional triplet sensitization pathways become available from coupled radical-sensitizer molecules (TTM-1Cz-DPA) compared to radical only (TTM-1Cz) (see Figures S4 and S5, eqs S1–S4).

The Dexter transfer directly from ^2^[D_1_S_0_] (DX1) requires overlap of the radical SOMO and annihilator LUMO and overlap of radical HOMO and annihilator HOMO simultaneously (see eq S1.1). This is unlikely since the radical HOMO and SOMO are on different parts of the molecule – in TTM-1Cz-DPA DFT calculations show that the radical SOMO is mainly localized on TTM and the radical HOMO on carbazole (Figure S7). Moreover, in none of the relaxed structures of the Rad-DPA:DPA and DPA:Rad-DPA complexes obtained from computation (Figure S8) is the annihilating DPA close to both TTM and carbazole simultaneously – in the lowest energy dimer structure the DPA annihilator and radical moiety are actually far separated. These orbitals combined with the molecular orientation of the dimers will likely result in a diminishingly small Dexter integral.

The Dexter transfer via ^2,4^[D_0_T_1_] (DX2 and DX3) instead requires simultaneous overlap of the annihilator HOMO and acene HOMO, and of the annihilator LUMO and acene LUMO (see eqs S1.2 and S1.3). This is more likely since the acene HOMO and LUMO are in similar places in space as are the annihilator HOMO and LUMO in the three lowest-energy Rad-DPA:DPA structures. Consequently, orbital interaction arguments predict that the most likely Dexter transfer mechanism for triplet formation on the annihilator is via the ^2,4^[D_0_T_1_] state on the sensitizer and not directly from ^2^[D_1_S_0_] (Figure S4: DX2-DX3 and eqs S1.2–S1.3).

The intermolecular triplet sensitization efficiency in radical sensitizer:annihilator systems was tested by Stern–Volmer studies. Here the kinetics of HTTM-1Cz-DPA sensitization with TTM-1Cz-DPA was characterized by monitoring fluorescence lifetime quenching of the radical by the annihilator (Figure 3e, dark blue markers). Quenching by the DPA-annihilator is also given for comparison. An intermolecular quenching constant of 2.85 × 10^8^ M^–1^ s^–1^ and 3.65 × 10^8^ M^–1^ s^–1^ was determined between TTM-1Cz-DPA with HTTM-1Cz-DPA and DPA, respectively. This value is lower than the diffusion-controlled limit in solution (10^10^ M^–1^ s^–1^) and reflects a low free energy difference in energy transfer from the TTM-1Cz-DPA doublet/quartet excited state manifold. DPA has a slightly higher quenching rate that may be attributed to lower steric clash on intermolecular energy transfer compared to HTTM-1Cz-DPA. An analogous measurement using the BPEA annihilator showed a higher quenching constant of 8.93 × 10^8^ M^–1^ s^–1^ (see Figure S18, green markers). The increased quenching arises from the lower T_1_ of BPEA (1.4 eV)^49^ versus DPA (1.8 eV)_._ ^49,50^ Overall we can evaluate the intermolecular energy transfer efficiency values of Φ_IET_ = 43% for TTM-1Cz-DPA:HTTM-1Cz-DPA where [HTTM-1Cz-DPA] = 10 mM; Φ_IET_ = 49% for TTM-1Cz-DPA:DPA where [DPA] = 10 mM; and Φ_IET_ = 66% for TTM-1Cz-DPA:BPEA where [BPEA] = 8 mM. The coupled radical-annihilator with extended excited-state lifetime presents a significant improvement in sensitization efficiency compared to TTM-1Cz alone.

### 2.4 Photochemical Upconversion from TTM-1Cz-DPA and HTTM-1Cz-DPA

Having seen that the energy transfer pathways for upconversion are allowed theoretically, and that intermolecular triplet sensitization is efficient in these systems, we now consider photochemical upconversion of TTM-1Cz-DPA with its closed-shell counterpart HTTM-1Cz-DPA. A toluene solution of TTM-1Cz-DPA (50 μM) and HTTM-1Cz-DPA (10 mM) was irradiated with continuous 658 nm light (1.88 eV) (Figure 4). The TTM-1Cz-DPA:HTTM-1Cz-DPA sample shows an upconverted emission peak at 450 nm (2.76 eV) where the PL was detected using a 650 nm short-pass filter. This emission is attributed to HTTM-1Cz-DPA fluorescence, corresponding to an upconversion energy shift of 0.88 eV.

**Figure 4:**
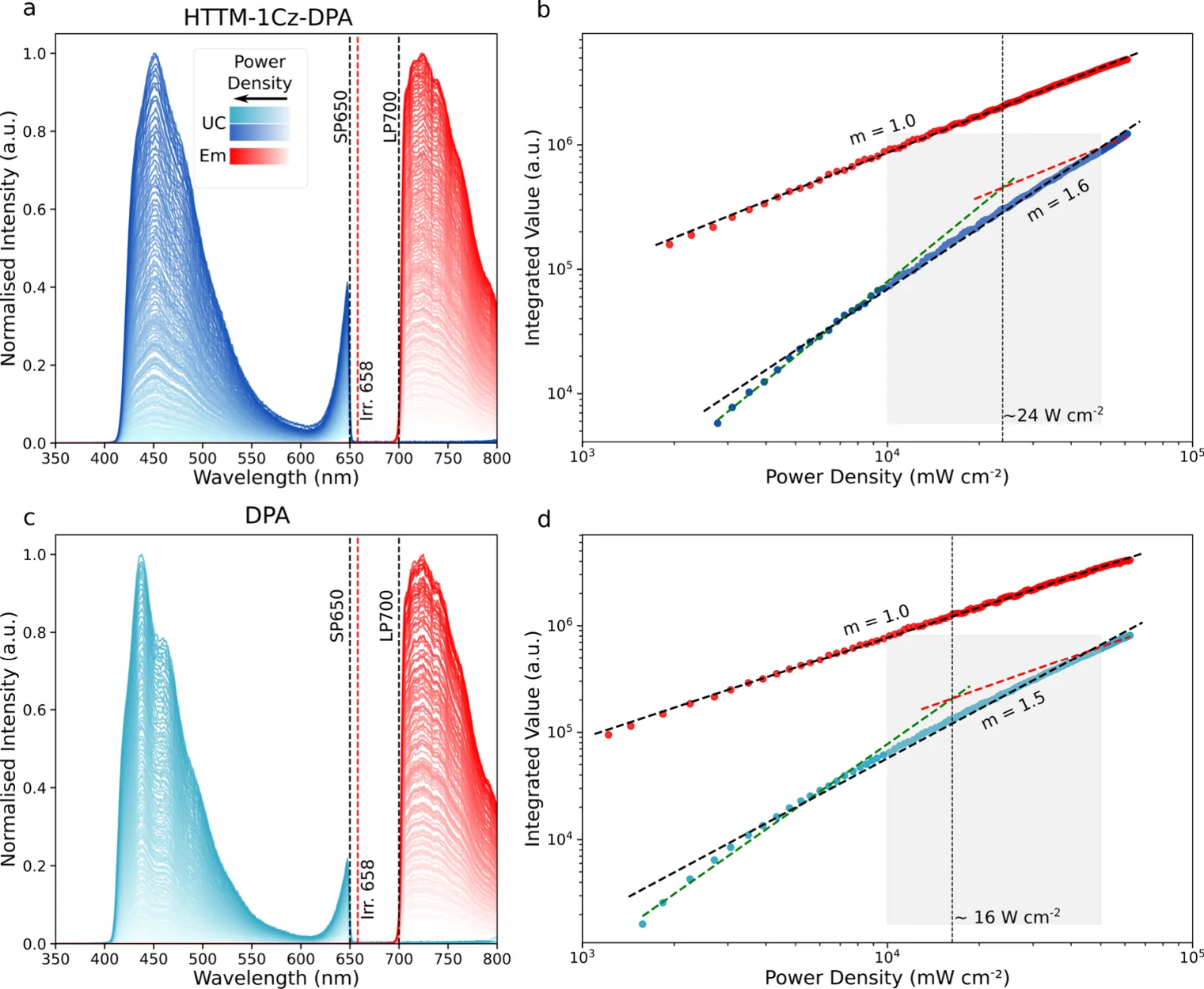
(a, c) Normalized emission spectra for TTM-1Cz-DPA (50 μM) and (a) HTTM-1Cz-DPA (10 mM) or (c) DPA (10 mM) in toluene, showing TTM-1Cz-DPA emission (red, 700 nm long-pass filter) and upconverted HTTM-1Cz-DPA or DPA emission (dark blue/light blue, 650 nm short-pass filter). (b, d) Power density dependence of TTM-1Cz-DPA emission (red markers, 700–800 nm) and upconverted emission of TTM-1Cz-DPA with (b) HTTM-1Cz-DPA (dark blue markers, 400–600 nm) and (d) DPA (light blue markers, 400–600 nm) as annihilators. The gradient of a linear fit, *m*, is given for both emission ranges. Quadratic and linear fits (green and red respectively) are shown and are for reference only. Data points were excluded between 10 and 40 mW cm^–2^ to fit representative ‘2/1’ gradients.

Our transient absorption data of TTM-1Cz-DPA following 400 nm excitation shows that ^2^[D_0_S_1_] is short-lived (<20 ps, Figure S3) and outcompetes radiative decay from this state. This suggests that upconverted emission from TTM-1Cz-DPA:HTTM-1Cz-DPA likely follows from triplet–triplet annihilation of HTTM-1Cz-DPA. Single-component upconversion where TTM-1Cz-DPA acts as sensitizer and annihilator appears unviable from our studies.^33^ Upconversion with TTM-1Cz-DPA:DPA mixture is also studied. In TTM-1Cz-DPA:DPA, the upconverted emission at 437 nm (2.84 eV) is observed and corresponds to an energy shift of 0.96 eV. These results show that by exchange of the radical from ‘C·’ in TTM-1Cz-DPA to the hydrogenated equivalent ‘C–H’ in HTTM-1Cz-DPA, the material can be switched from sensitizer to annihilator character. In HTTM-1Cz-DPA, the photophysics reflects the DPA moiety and its singlet–triplet excited states.

For comparison, a lower energy annihilator was also investigated, TTM-1Cz-DPA (50 μM):BPEA (8 mM), with results given in the Supporting Information (Figure S19). Following 658 nm excitation, we observed an emission peak at 510 nm, corresponding to BPEA fluorescence following upconversion with an energy shift of 0.55 eV.

Figure 4b, 4d show the relationship between the power density of irradiation and the upconverted emission intensity (dark and light blue markers) for the TTM-1Cz-DPA:HTTM-1Cz-DPA and TTM-1Cz-DPA:DPA solutions, respectively. From the gradient, we see at low power densities a quadratic dependence of the upconverted emission on the power density, which reflects bimolecular kinetics from TTA.^51^ The emission from the radical sensitizer follows a linear power dependence (red markers) that suggests no significant involvement in these kinetics, for example, by energy transfer of upconverted singlet states of the annihilator to TTM-1Cz-DPA.^24^

For both TTM-1Cz-DPA:HTTM-1Cz-DPA (Figure 4b) and TTM-1Cz-DPA:DPA (Figure 4d) we do not observe the expected transition from quadratic to linear power dependence on upconversion intensity. This is shown by unsatisfactory slope 2:1 (red and green) fit and a best fit line (black line), which do not capture all the data points for log-scale plots of integrated upconversion intensity vs power density. The threshold intensity for transition between quadratic and linear regimes is underestimated by this analysis (>24 W cm^–2^ for TTM-1Cz-DPA:HTTM-1Cz-DPA and >16 W cm^–2^ for TTM-1Cz-DPA:DPA). The incomplete transformation to linear behavior suggests that reverse energy transfer of the annihilator to the sensitizer competes with upconversion, as expected in the design of systems that maximize the energy shift.^52,53^ This limitation means we cannot apply standard kinetic modeling of photochemical upconversion to determine the threshold intensity, as it assumes no intermolecular energy back transfer following triplet sensitization.^54^ Larger energy offset between the ^2,4^[D_0_T_1_] and the ^3^[T_1_] of the annihilator (see Figure S19) can mitigate this and lower the threshold intensity, but will also reduce the upconversion energy shift. We note this trade-off between maximizing upconversion energy shift and efficiency is present in our system.

For the open- and closed-shell sensitizer:annihilator pair of TTM-1Cz-DPA:HTTM-1Cz-DPA, we find a maximum upconversion efficiency of Φ_UC_ = 7 ± 2% at 62 W cm^–2^ excitation density. We also highlight a substantial improvement in the measured maximum upconversion efficiency using the same DPA annihilator with TTM-1Cz-DPA as the sensitizer (Φ_UC_ = 12 ± 4%) compared to TTM-1Cz (Φ_UC_ = 0.13%). Continuous irradiation studies (6 W cm^–2^, Figure S20) of samples in toluene support the high stability of TTM-1Cz-DPA and the upconverted emission from TTM-1Cz-DPA:HTTM-1Cz-DPA samples in solution (<1% change over 8 h). We have also demonstrated upconversion using luminescent organic radicals in the solid state for the first time (see Supporting Information section 8). Films were prepared in a rigid PMMA matrix and showed Φ_UC_ of ∼0.001% and ∼0.0002% for TTM-1Cz-DPA combined with HTTM-1Cz-DPA and DPA, respectively. The higher observed Φ_UC_ for HTTM-1Cz-DPA sample may indicate better compatibility of sensitizer and annihilator in processing films using components from the same molecular frame, despite higher upconversion performance reported for TTM-1Cz-DPA:DPA in solution. In future work, different processing methods will be explored to study and optimize morphology effects on triplet diffusion, concentration quenching and upconversion loss from back transfer (^1^[S_1_] + ^2^[D_0_S_0_] → ^1^[S_0_] + ^2^[D_1_S_0_]), which can limit performance in the solid state.

Upconversion following intermolecular annihilation of two [D_0_T_1_] states to a higher energy and emissive [D_0_S_1_] with radical-annihilator dyads was previously suggested.^33^ The proposed mechanism (Figure 1b) is formally anti-Kasha in nature as doublet emission from ^2^[D_0_S_1_] must outcompete with internal conversion to lower-lying doublet excited states such as ^2^[D_1_S_0_]. Here the mechanism was tested by preparing samples of 50 μM, 1 mM and 10 mM TTM-1Cz-DPA in toluene, however no upconversion was observed following 661 nm irradiation at 62 W cm^–2^. We consider that the presence of hydrogenated precursor (HTTM-1Cz-DPA) of the radical-annihilator dyad in these samples may be necessary for efficient upconversion. In order to test this, we prepared new samples with 0.01:0.99, 0.10:0.90, 0.50:0.50 and 0.90:0.10 mixtures of precursor:radical (HTTM-1Cz-DPA:TTM-1Cz-DPA) at 50 μM, 1 mM and 10 mM total concentrations (Figure S23, Table S9). Upconversion could not be detected for the 50 μM samples. Within the 1 mM samples, samples containing a 0.90:0.10 ratio of HTTM-1Cz-DPA to TTM-1Cz-DPA showed upconversion (Φ_UC_ ≈ 0.02%). For 10 mM samples, upconversion was detected for 0.9:0.10 and 0.50:0.50 mixtures. The observation of upconversion depends on the total concentration of the samples, as well as the ratio of precursor to radical. This may be attributed to competing concentration effects of the radical sensitizer and annihilator components in upconversion. A higher concentration of the hydrogenated precursor that acts as the annihilator (HTTM-1Cz-DPA) will result in higher intermolecular energy transfer yield (Φ_IET_) for maximizing the overall upconversion efficiency – see modeling in Figure S24. We note that higher concentration of the radical sensitizer (TTM-1Cz-DPA) can lead to excited-state self-quenching, which leads to lower Φ_IET_ and lower upconversion efficiency. Our findings highlight the possibility of 'pseudo-single' component mixtures for photochemical upconversion from open-shell and closed-shell roles of sensitizer and annihilator based on the same molecular frame.

## 3 Conclusion

Here we consider how the exchange of ‘C•’ to ‘C–H’ in the trityl moiety of a linked radical-annihilator molecule modifies the energy level structure from efficient open-shell sensitizer to closed-shell annihilator for photochemical upconversion. We use TTM-1Cz-DPA as a model radical-annihilator molecule, in which doublet–triplet energy degeneracy of its constituent parts leads to reversible intramolecular energy transfer and extended radical excited-state lifetime (>200 ns) within a coupled doublet/quartet excited state manifold: ^2^[D_1_S_0_] ↔ ^2,4^[D_0_T_1_]. The extended lifetime and generation of ‘preformed’ triplets are attractive sensitizer properties of linked radical-annihilator molecules compared to radical only (TTM-1Cz). The hydrogenated counterpart, HTTM-1Cz-DPA, behaves like the classical DPA annihilator. We find efficient red (658 nm) to blue (450 nm/437 nm) photon upconversion in intermolecular systems using TTM-1Cz-DPA as the sensitizer paired with HTTM-1Cz-DPA and DPA as annihilator components, leading to apparent anti-Stokes energy shifts of 0.9 eV with 7% and 12% quantum efficiency. The molecular structures and physiochemical properties of the energy upconversion components in open-/closed-shell pairs are inherently matched, potentially enabling better compatibility for materials processing and novel optoelectronic devices that exploit low energy electrical excitation of organic radicals for higher energy emission.^55^

## 4 Methods

### 4.1 Steady-State Photoluminescence (PL)

Steady-state photoluminescence spectra and absolute quantum yields were measured using a Hamamatsu Quantaurus-QY Plus UV-NIR absolute PL quantum yield spectrometer (C13534-11).

### 4.2 UV–vis

UV–vis absorption spectra were recorded with a PerkinElmer Lambda 365+ double-beam UV–visible spectrophotometer.

### 4.3 Upconversion (UC)

Upconversion spectra were acquired using an Oxford Instruments Kymera 328i spectrograph with an Andor Newton 920 CCD camera. Excitation was provided by either a 520 or 658 nm laser photodiode (ThorLabs L520P50 or L660P120). The emission was spectrally filtered to remove the laser excitation using long/short-pass filters (ThorLabs FESH0550/600/650 FELH 550/700). Samples were prepared in toluene and oxygen excluded using oxygen scavenger (bis­(methylthio)­methane) reported by Dzebo et al.^56^

The relative quantum yield of upconversion was calculated using the following equation:

1 $$\Phi_{UC}=\Phi_{R}\cdot \left(\frac{I_{UC}}{I_{R}}\right)\left(\frac{1-T_{R}}{1-T_{UC}}\right)$$

where Φ_*UC*_ and Φ_*R*_ are the quantum yields of upconversion and emission from a reference sample (TTM-1Cz, measured using an absolute quantum yield spectrometer as described above), and *I*_*UC*_ and *I*_*R*_ are the integrated intensities of the upconversion emission spectra and the emission spectra of a reference sample. *T*_*R*_ and *T*_*UC*_ are the transmittances of the reference and the upconversion samples at the photoexcitation wavelength, respectively. For solid-state samples, an integrating sphere method using the same spectrometer and laser system was used to determine Φ_*UC*_.

The power density in upconversion studies was modulated by using a variable neutral-density wheel. The upconversion signals were captured using either a 600 nm or a 550 nm short-pass filter. The TTM-1Cz-DPA emission was captured using a 700 nm long-pass filter. Either a linear or piecewise-linear (2 slopes with a breakpoint) model was used to fit the data. The Akaike information criterion (AIC) was used to justify the choice of the fit.

### 4.4 Transient Absorption (TA) and Transient Photoluminescence (TrPL)

From a 1030 nm Yb:KGW 25 kHz laser source (Pharos, Light Conversion), pump excitation was generated using a nonlinear optical parametric amplifier (OPA) capable of generating femtosecond laser pulses between 300 and 2700 nm (Orpheus + Lyra, Light Conversion). For TA, measurements were carried out using a fsTA setup (HARPIA-TA, Light Conversion). Visible (510–950 nm) and UV (400–490 nm) probe pulses were generated through irradiation of a sapphire crystal with 1030 and 515 nm radiation, respectively. A 50 Hz chopper was used, and the temporal resolution is achieved using a delay stage with a maximum delay of 8 ns. For TrPL, a Hamamatsu C10910 Streak camera was used, which utilizes a Princeton HRS-300 Spectrometer and a Hamamatsu C1097-05 delay unit. Spectra were acquired by operating in analogue integration mode. A 500 ns and 2 μs window were acquired and joined using least-squares scaling of the overlapping data.

For Stern–Volmer measurements TrPL spectra were collected using an Edinburgh Instruments FLS1000 spectrometer. The photoexcitation was provided by a 635 nm pulsed laser photodiode (Edinburgh Instruments EPL-635, 65 ps pulse width). A window of ±15 nm was set for the emission wavelength, and the acquisitions were made in ‘reverse’ mode, collecting until a peak number of counts was reached.

### 4.5 Electron Spin Resonance (ESR)

All measurements were performed at the X-band (9.75 GHz) in quartz ESR tubes with an outer diameter of 3.8 mm and an inner diameter of 3 mm. Before measurements, all sample solutions were degassed on a Schlenk line using the freeze–pump–thaw technique, after which the tubes were flame-sealed.

#### 4.5.1 Continuous Wave (cw) ESR Spectroscopy

The cw ground state spectrum was recorded at room temperature on a Bruker EMXnano benchtop ESR spectrometer. The modulation frequency was set to 100 kHz, and the modulation amplitude was set to 0.1 mT at a microwave power of 0.398 mW (24 dB). The recorded spectrum was background-corrected, frequency-corrected to 9.75 GHz, and field-corrected using a carbon fiber standard with *g* = 2.002644.^57^

#### 4.5.2 Transient Continuous Wave ESR Spectroscopy

TrESR was recorded on a Bruker ELEXSYS E580 spectrometer, equipped with a Bruker ER 4118X-MD5 resonator. Cryogenic experiments were performed at a constant temperature of 80 K, using an Oxford Instruments nitrogen gas-flow cryostat (CF 935). The samples were excited directly with depolarized laser light through the optical window of the cryostat and resonator, using the wavelength of 600 nm and the excitation energy of about 2.3 mJ at a repetition rate of 50 Hz (∼5 ns pulse duration). The spectra were acquired in direct detection mode with a video amplifier bandwidth of 20 MHz using the built-in transient recorder and a microwave power of 1.5 mW (20 dB). Thus, any positive signal corresponds to an absorptive transition (a) and any negative signal to an emissive (e) one. The transient recorder was used in AC-AFC mode, and the transient signal was fed through a low-noise voltage preamplifier (Stanford Research Systems SR560) with a bandpass filter of 3 kHz to 1 MHz before entering the detection circuitry. A time trace with 4096 points and a time base of 4 ns was recorded for every magnetic field value. The acquired 3D data sets were baseline-corrected in both dimensions, time and field, using a lab-written MATLAB routine. All trESR spectra were frequency-corrected to 9.75 GHz and field-corrected using a carbon fiber standard with *g* = 2.002644.^57^

### 4.6 Quantum Chemical Calculations

Doublet ground state geometries of TTM-1Cz-DPA and TTM-1Cz-DPA:DPA dimers were optimized in the framework of unrestricted density functional theory (DFT) using the ωB97X-D exchange-correlation functional and the 6–31g­(d,p) basis set.

Vertical transition energies were computed using time-dependent DFT within the Tamm–Dancoff approximation^58^ (TDA-DFT) at the CAM-B3LYP/6–311g­(d,p) level. Excited state analysis was performed with the TheoDORE program.^59^ Electronic couplings between the relevant excited states were evaluated within the Fragment Excitation Difference (FED) scheme. In this two-state approach, the system is partitioned into two fragments, the donor and the acceptor, and excitation-localized states are obtained by maximizing the excitation number difference between the donor and the acceptor, and the couplings given as

$$V_{ij}^{FED}=\frac{\Delta E_{ij}{\overset{®}{\Delta x}}_{ij}}{\sqrt{{\left(\Delta x_{ii}-\Delta x_{jj}\right)}^{2}+4{\overset{®}{\Delta x}}_{ij}^{2}}}$$

with Δ*E*_*ij*_ the energy difference between adiabatic states *i* and *j*, Δ*x*_*ii*_, Δ*x*_*jj*_ and ${\overset{®}{\Delta x}}_{ij}$ the donor–acceptor difference in excitation number (the latter being computed as the integral of the difference between the detachment and attachment densities) for states *i* and *j*, as well as the transition between *i* and *j*, respectively. The excitation number corresponds to a population of the eigenvectors of the difference density operator defined as the difference between the densities of the *i*-th excited states and the ground state. The bar refers to symmetrized quantities since Δ*x*_*ji*_ and Δ*E*_*ji*_ are likely not identical.

For TTM-1Cz-DPA, electronic couplings were also evaluated via a diabatization procedure using the Boys^60^ localization scheme, where the adiabatic states were computed at the restricted active-space configuration interaction (RASCI)^61^ method and the def2-SVP basis set. The RAS-CI calculation was carried out with one-hole and one-particle contributions and a DFT reference configuration (CAM-B3LYP/def2-SVP), considering a RAS2 space with five electrons in five orbitals. This approach produces spin-pure states (no spin contamination) and was shown in a previous study to provide reliable results for a system of similar nature^32^ and constitutes a reference for validating the FED couplings.

All calculations were done using Gaussian 16,^62^ ORCA,^63^ and Q-Chem^64^ program packages. The DrawMol program was used for visualization of the natural transition orbitals.^65^

### 4.7 Preparation of Solid-State Samples

Upconversion films were produced by drop-casting 75 μL of solution containing PMMA (200 mg mL^–1^, 101,000 g mol^–1^), 100 μM of TTM-1Cz-DPA sensitizer and 20 mM of either DPA or HTTM-DPA annihilator in chloroform. Samples were encapsulated under an inert atmosphere using a quartz coverslip and UV-curable resin.

## Supplementary Material



## Data Availability

The data that support the findings of this study are available in the following repository: DOI: 10.5281/zenodo.20345529

## Notes

Experimental and synthetic details, additional spectroscopic analysis, theoretical calculations and modeling. (PDF)
