# Supplementary material for: Open- and Closed-Shell Roles of Sensitizer and Annihilator in Pseudo-Single Component Mixtures for Upconversion
Source: J Am Chem Soc. 2026 Jul 3;148(33):35375–85. doi: 10.1021/jacs.6c04090 (PMC13523683; doi:10.1021/jacs.6c04090)
Supplement: Supplementary file 1 [file ja6c04090_si_001.pdf]

Supplementary Materials for

Open- and Closed Shell Roles of Sensitizer and Annihilator in Pseudo-Single Component Mixtures for Upconversion

Kieran D. Richards[1,2±], Wenzhao Wang[3±], Philipp Thielert[4], James D. Green[5], John M. Hudson[1,2], Claire Tonnelé[6,7], David Casanova[6,7], Yoann Olivier[8], Timothy J. H. Hele[5], Sabine Richert[4], Feng Li[3], Emrys W. Evans[1,2*]*

[1]Department of Chemistry, Swansea University, Singleton Park, Swansea, SA2 8PP, United Kingdom

[2]Centre for Integrative Semiconductor Materials, Swansea University, Fabian Way, SA1 8EN, United Kingdom

[3]State Key Laboratory of Supramolecular Structure and Materials, College of Chemistry, Jilin University, Qianjin Avenue 2699, Changchun, 130012, P. R. China

[4]Institute of Physical Chemistry, University of Freiburg, Albertstraße 21, 79104, Freiburg, Germany

[5]Department of Chemistry, University College London, WC1H 0AJ, London, UK

[6]Donostia International Physics Centre, 20018 Donostia, Euskadi, Spain

[7]Ikerbasque, Basque Foundation for Science, 48009 Bilbao, Euskadi, Spain

[8]Laboratory for Computational Modelling of Functional Materials, Namur Institute of Structured Matter, University of Namur, Rue de Bruxelles 61, 5000 Namur, Belgium

[±]K.D.R. and W.W. contributed equally to this work

*Corresponding authors: lifeng01@jlu.edu.cn (F. L.); emrys.evans@swansea.ac.uk (E. W. E)

1. Synthesis and characterisation

All chemical reagents and solvents were purchased from commercial suppliers and used without further purification (unless otherwise stated). Tetrahydrofuran (THF) was distilled before used. Column chromatography was performed with silica gel (200-300 mesh).

The ^{1}H nuclear magnetic resonance (NMR) spectra were recorded with a Bruker AVANCE-III 500 NMR spectrometer for samples in deuterated Dimethyl sulfoxide (DMSO) at ambient temperature. MALDI-TOF mass spectra were recorded on a Bruker Autoflex Speed TOF/TOF mass spectrometer with DCTB matrix.

Elemental analysis (EA) was performed on a Elementar Vario micro cube elemental analyser.

HTTM and TTM were prepared according to our previous report (Scheme 1).[1] A general procedure for the synthesis of radical TTM-1Cz-DPA is given in Scheme 2.

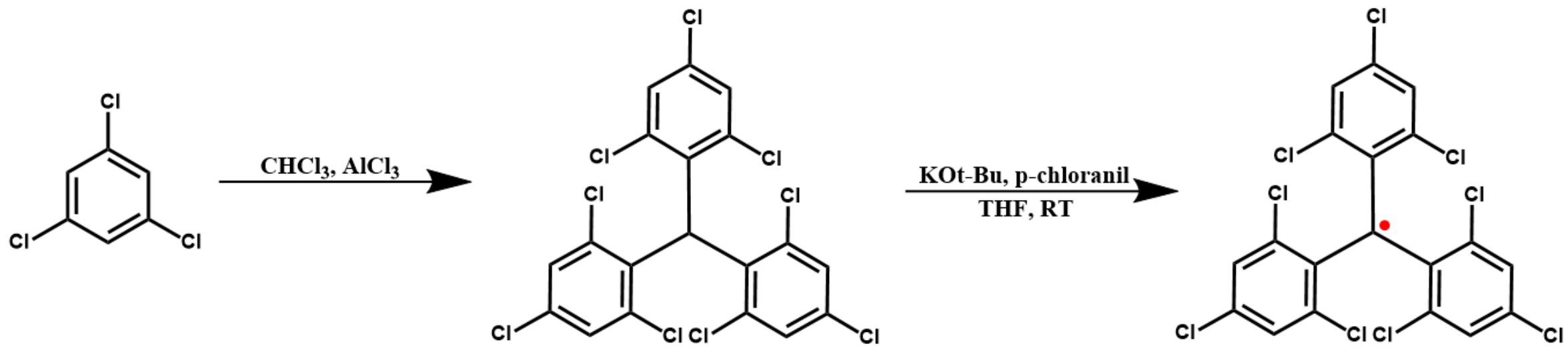

Scheme 1. *Synthesis of HTTM and TTM*

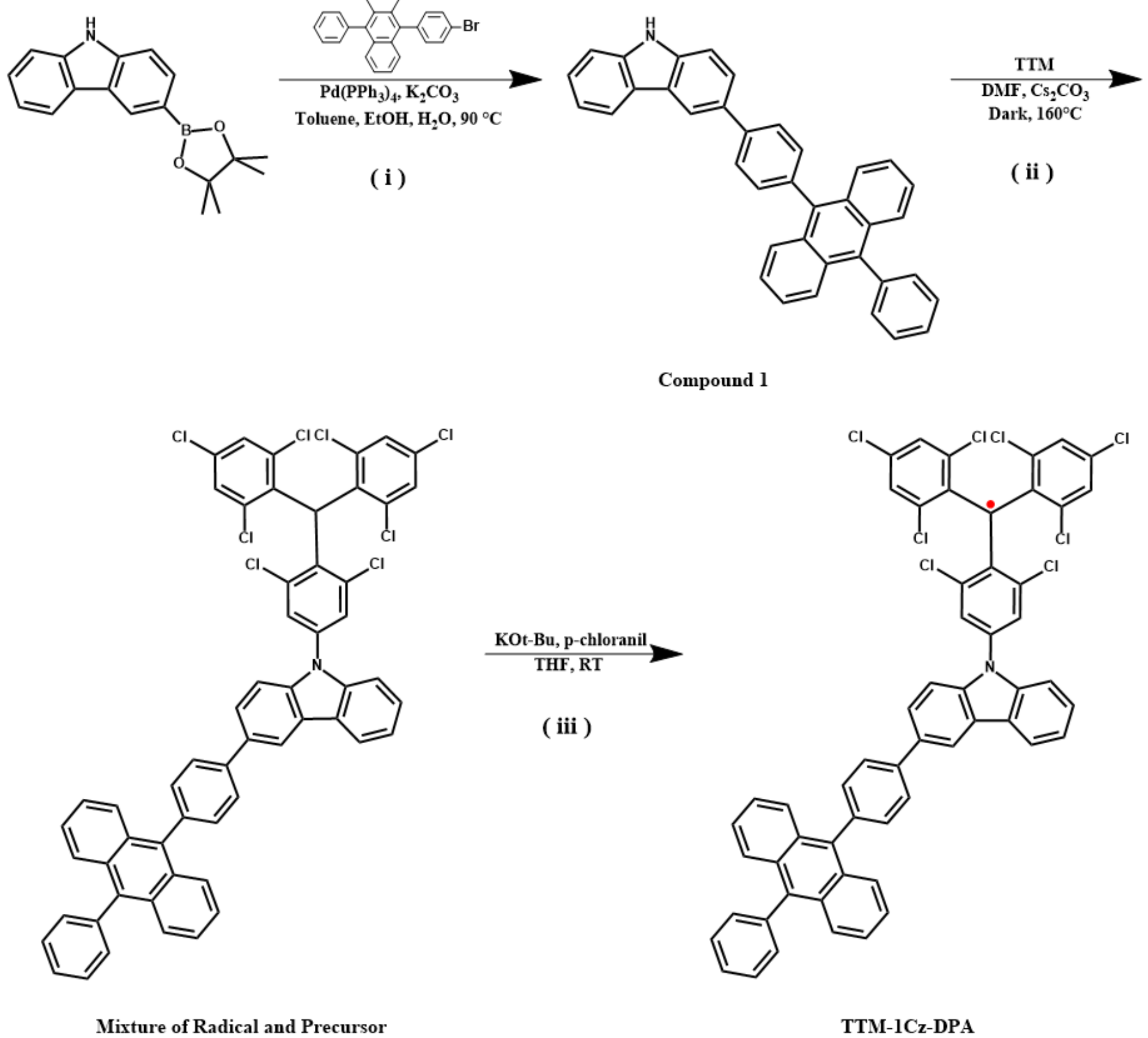

Scheme 2. *Synthesis of TTM-1Cz-DPA*

Scheme 2. (i): 3-(4,4,5,5-tetramethyl-1,3,2-dioxaborolan-2-yl)carbazole (3.0 mmol) and 9-(4-bromophenyl)-10-phenylanthracene (2.0 mmol) were dissolved in a mixed solvent of toluene (24 mL), K_2CO_3 aqueous solution (16 mL, 2 M) and ethanol (8 mL), then catalyst $Pd(PPh_3)_4$ (0.1 mmol) was added under argon atmosphere. The mixture was stirred at 90°C for 48 h under argon atmosphere and in the dark. Then the solution was cooled to room temperature and filtered. Reprecipitation from dichloromethane-petroleum ether system yielded compound 1 as yellow powder.

Compound 1:yellow powder(0.90 g, 91%) ; ^{1}H NMR (500 MHz, DMSO-d_6) δ 11.40 (s, 1H), 8.67 (d, *J* = 1.9 Hz, 1H), 8.28 (d, *J* = 7.7 Hz, 1H), 8.08 (d, *J* = 7.8 Hz, 2H), 7.92 (dd, *J* = 8.5, 1.9 Hz, 1H), 7.79 – 7.41 (m, 18H), 7.22 (t, *J* = 7.4 Hz, 1H).

MALDI-TOF-MS (m/z): calculated for $C_{38}H_{25}N$, 495.63. Found, 495.47 (Fig. S1).

EA: calculated for $C_{38}H_{25}N$: C 92.09, H 5.08, N 2.83. Found: C 92.02, H 4.92, N 2.75.

Scheme 2. (ii): TTM (3.0 mmol), compound 1 (2.0 mmol), anhydrous Cs_2CO_3 (3.0 mmol) were added in DMF (50 ml). The mixture was stirred at 160°C for 12 h under argon atmosphere and dark conditions. After the reaction mixture was cooled to room temperatures and poured into (0.2 M) hydrochloric acid solution; the precipitate was filtered and washed with water three times. The crude product was dissolved in dichloromethane and extracted with water and dichloromethane. The organic layer was dried over $MgSO_4$ and evaporated under vacuum. The crude product was purified by silica gel column chromatography (using petroleum ether: dichloromethane = 5:1, v/v). Mixtures of TTM-1Cz-DPA and its precursor compounds were obtained.

TTM-1Cz-DPA and its precursor: light green powder (0.56 g, 28%)

Scheme 2. (iii): Under argon atmosphere and dark conditions, the KOt-Bu (5.0 mmol) was added to the THF solution of TTM-1Cz-DPA and its precursor mixtures (0.5 mmol); the solution was stirred for 5 h at room temperature. Then tetrachloro-p-benzoquinone (2.5 mmol) was added and the solution was stirred for another 2 h. After the reaction finished, the solvent was removed under vacuum, and the crude product was purified by silica gel column chromatography (using petroleum ether: dichloromethane = 5:1, v/v). The desired TTM-1Cz-DPA was obtained.

TTM-1Cz-DPA: dark green powder (0.35 g, 69%)

MALDI-TOF-MS (m/z): calculated for $C_{57}H_{30}Cl_8N$, 1012.47. Found, 1010.35 (Fig. S2).

EA: calculated for $C_{57}H_{30}Cl_8N$: C 67.62, H 2.99, N 1.38. Found: C 68.19, H 3.50, N 1.40.

Mass Spectrometry

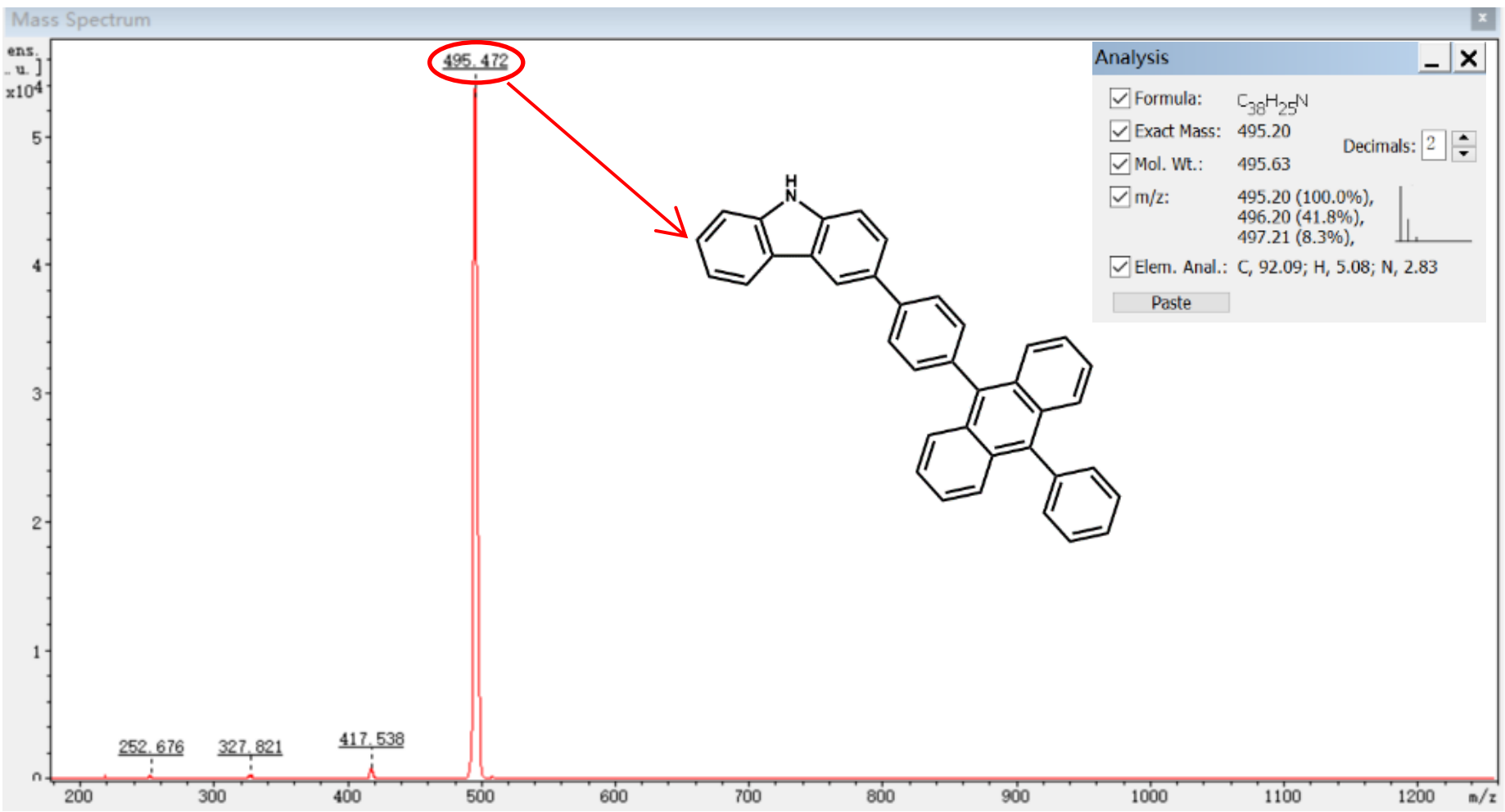

Figure S1. *Mass Spectrum of Compound 1*

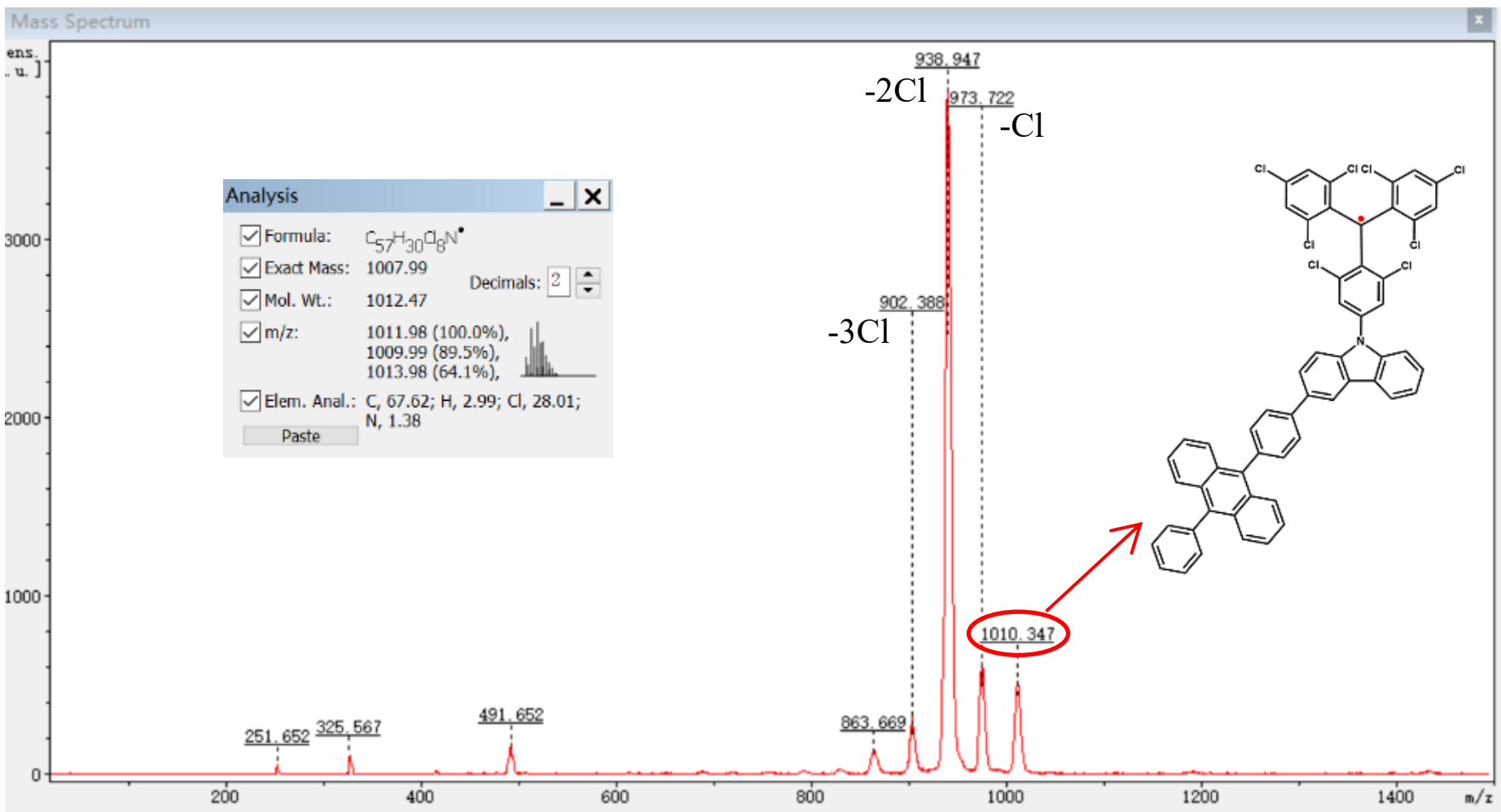

Figure S2. *Mass Spectrum of TTM-1Cz-DPA*

2. Transient absorption spectroscopy

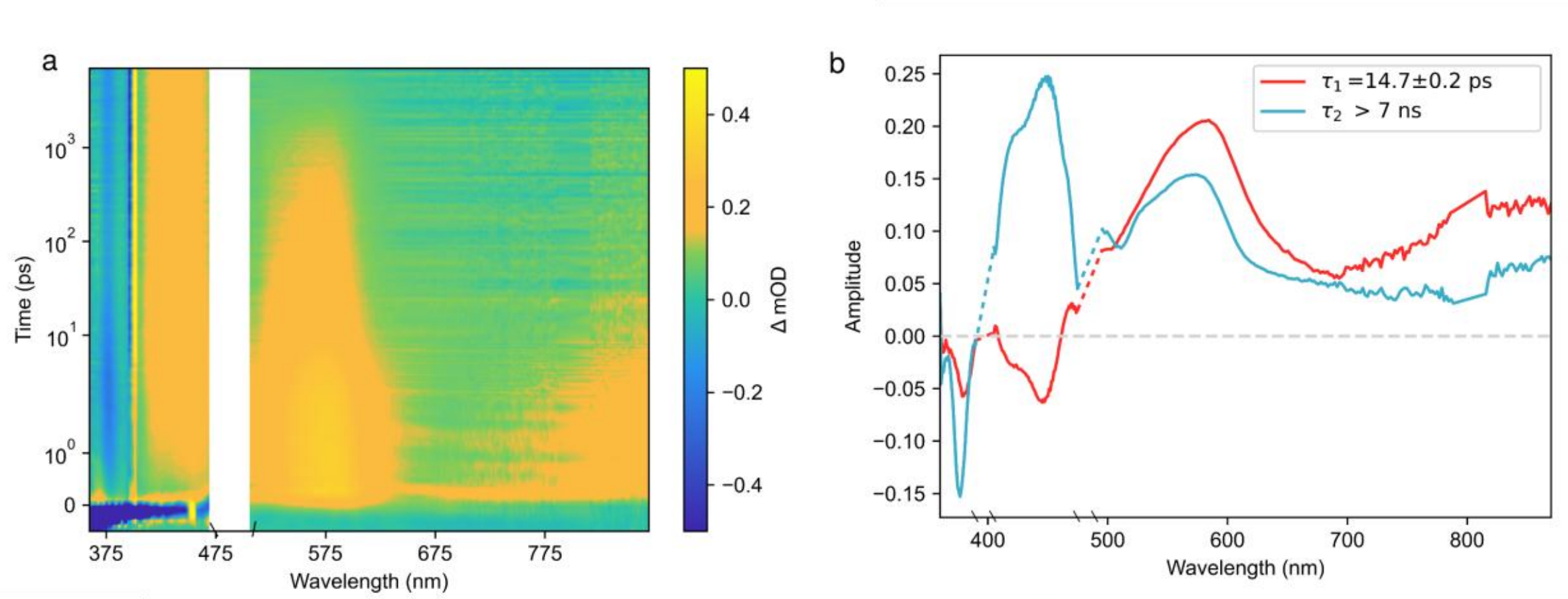

***Figure S3. a** Femtosecond transient absorption spectra of TTM-1Cz-DPA following 400 nm excitation (1 mJ cm^{-2}). **b** Decay associated spectra obtained by global analysis, showing two time constants at 14.7 ps and >7 ns, longer than the instrument collection window. Axes breaks are present between probe regions and around 400 nm (laser scatter). These regions are indicated by dashed lines.*

3. Quantum chemical theory and calculations

Electronic structure theory of energy transfer mechanisms

We have applied electronic structure theory to possible mechanisms for energy transfer from the sensitiser to a triplet state on the annihilator. To derive analytic expressions for energies and coupling terms of the relevant states involved, we apply electronic structure theory from recent work on radical and closed-shell systems.[2–10] We first examine the Dexter mechanisms from the $^2[D_1S_0]$ and $^{2/4}[D_0T_1]$ states. The mechanisms from $^{2/4}[D_0T_1]$ must first pass through $^2[D_1S_0]$ as this is the state initially produced on photoexcitation, and then internal conversion to $^2[D_0T_1]$ proceeds via a Dexter or charge-transfer state mediated mechanism. In general $^4[D_0T_1]$ can be generated from intersystem crossing from $^2[D_1S_0]$ or $^2[D_0T_1]$. As mentioned in the main text, this internal conversion to $^2[D_0T_1]$ takes place rapidly in TTM-1Cz-DPA, as seen previously in other doublet radical-triplet acene systems.[9] We use the following notation: R is for Radical (TTM-1Cz), A is for Acene (DPA in TTM-1Cz-DPA), N for annihilator (free DPA). Orbital 0 is the SOMO, 1 the HOMO and $1'$ the LUMO.

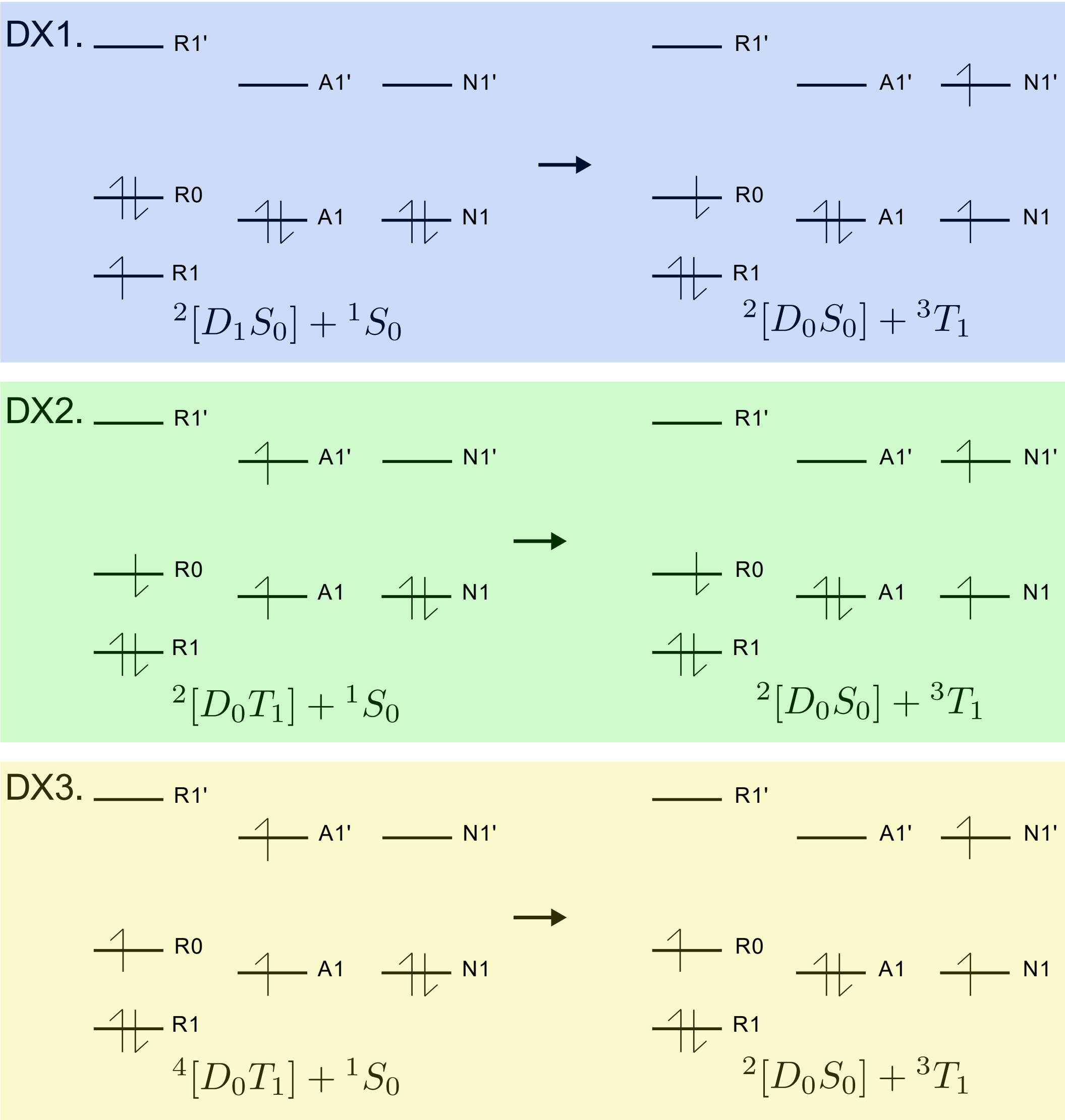

Figure S4. *Molecular orbital diagrams for Dexter mechanisms in energy transfer from radical-acene system to annihilator. 'R', 'A' and 'N' refer to orbitals on the radical, acene and annihilator respectively. Orbital 0 is the SOMO, 1 the HOMO and 1' the LUMO. Note that some states (the RHS of mechanism 1 and both sides of mechanism 2) are spin-adapted by including multiple spin configurations, however, for simplicity only a single determinant is shown in each MO diagram. The following algebra was derived using the spin-adapted states. Further details can be found in SI ref. 2 and 4.*

Theory for Dexter mechanism

The Hamiltonian mixing elements for each of the potential Dexter mechanisms are shown below, where ':' means 'has Hamiltonian mixing'.

Coupling terms for Dexter mechanisms:

$$^{2}[D_1S_0] + {}^{1}[S_0] \rightarrow {}^{2}[D_0S_0] + {}^{3}[T_1]: -\frac{3}{\sqrt{6}}(R0N1' \mid N1R1) \quad \text{S1.1}$$

$$^{2}[D_0T_1] + {}^{1}[S_0] \rightarrow {}^{2}[D_0S_0] + {}^{3}[T_1]: -(N1A1 \mid A1'N1') \quad \text{S1.2}$$

$$^{4}[D_0T_1] + {}^{1}[S_0] \rightarrow {}^{2}[D_0S_0] + {}^{3}[T_1]: -(N1A1 \mid A1'N1') \quad \text{S1.3}$$

$$^{3}[T_1] + {}^{1}[S_0] \rightarrow {}^{1}[S_0] + {}^{3}[T_1]: -(N1A1 \mid A1'N1') \quad \text{S1.4}$$

Where $(ij|kl)$ is a two-electron integral

Inspecting equations S1.1-S1.4 and Figure S4 we notice that all mechanisms are quantum mechanically allowed, and all are Dexter-like terms which, to be non-vanishing, require overlap between orbitals on the radical/acene and the annihilator. Mechanism DX1 (for $^{2}[D_1S_0]$, Equation S1.1) requires overlap of the radical SOMO and annihilator LUMO and overlap of annihilator HOMO and radical HOMO simultaneously. This is unlikely since the radical HOMO and SOMO are on different parts of the molecule. Analysing the molecular orbitals of TTM-1Cz-DPA shown in Figure S7, the radical SOMO (actually the lowest unoccupied natural transition orbital of the $^{2}[D_1S_0]$ state) is mainly localised on TTM and the radical HOMO (or the highest occupied natural transition orbital of the $^{2}[D_1S_0]$ state) on carbazole, as is also the case for TTM-1Cz.[5,10]

In none of the structures of the Rad-DPA:DPA and DPA:Rad-DPA complexes obtained from computation (Figure S8) is the annihilating DPA close to both TTM and carbazole simultaneously. Furthermore, in the lowest energy structure (dimer 3), the DPA annihilator and radical are far separated, minimising overlap of annihilator and radical orbitals.

The second and third mechanisms instead require simultaneous overlap of the annihilator HOMO and acene HOMO, and of the annihilator LUMO and acene LUMO. This is more likely since the acene HOMO and LUMO are in similar places in space as are the annihilator HOMO and LUMO in the three Rad-DPA:DPA complexes which are the lowest energy structures. Therefore, assuming a Dexter mechanism, it is more likely that the mechanism goes via $^{2/4}[D_0T_1]$ (mechanisms DX2 and DX3) rather than straight from $^{2}[D_1S_0]$ (DX1).

In fact, if the acene and annihilator are alternant hydrocarbons (DPA is both the acene and annihilator and is alternant), it follows from the theory of Coulson and Rushbrooke that the acene HOMO must be in the same region of space as the LUMO and likewise for the annihilator. Thus it is advantageous for the acene and annihilator to be alternant hydrocarbons (e.g. DPA) in Dexter transfer.[11]

Furthermore, we have shown that in the second and third mechanisms, the radical is essentially a 'spectator' in the energy transfer, and that the Hamiltonian mixing element is the same as for the energy transfer between isolated singlet and triplet states on the acene and annihilator respectively in Equation S1.4. This is confirmed by the very similar electronic couplings computed at RASCI level using Boys diabatization for the Rad-DPA:DPA complex and a DPA dimer that we used as a toy model to further rationalize the magnitude of this coupling (see 'Quantum Chemical Calculations' section below.

Theory for charge-transfer mechanism

Secondly, we examine the two-step mechanisms via charge-transfer (CT) states of which there are a total of six, shown in Figure S5. CT1-CT3 involve a CT state where the radical-acene part becomes an anion and the annihilator becomes a cation ($RA^{-}N^{+}$), whereas CT3-CT6 are the opposite ($RA^{+}N^{-}$). Again, the mechanisms via $^{2/4}[D_0T_1]$ involve the same additional step of internal conversion or intersystem crossing from $^{2}[D_1S_0]$ to $^{2/4}[D_0T_1]$ as mentioned earlier.

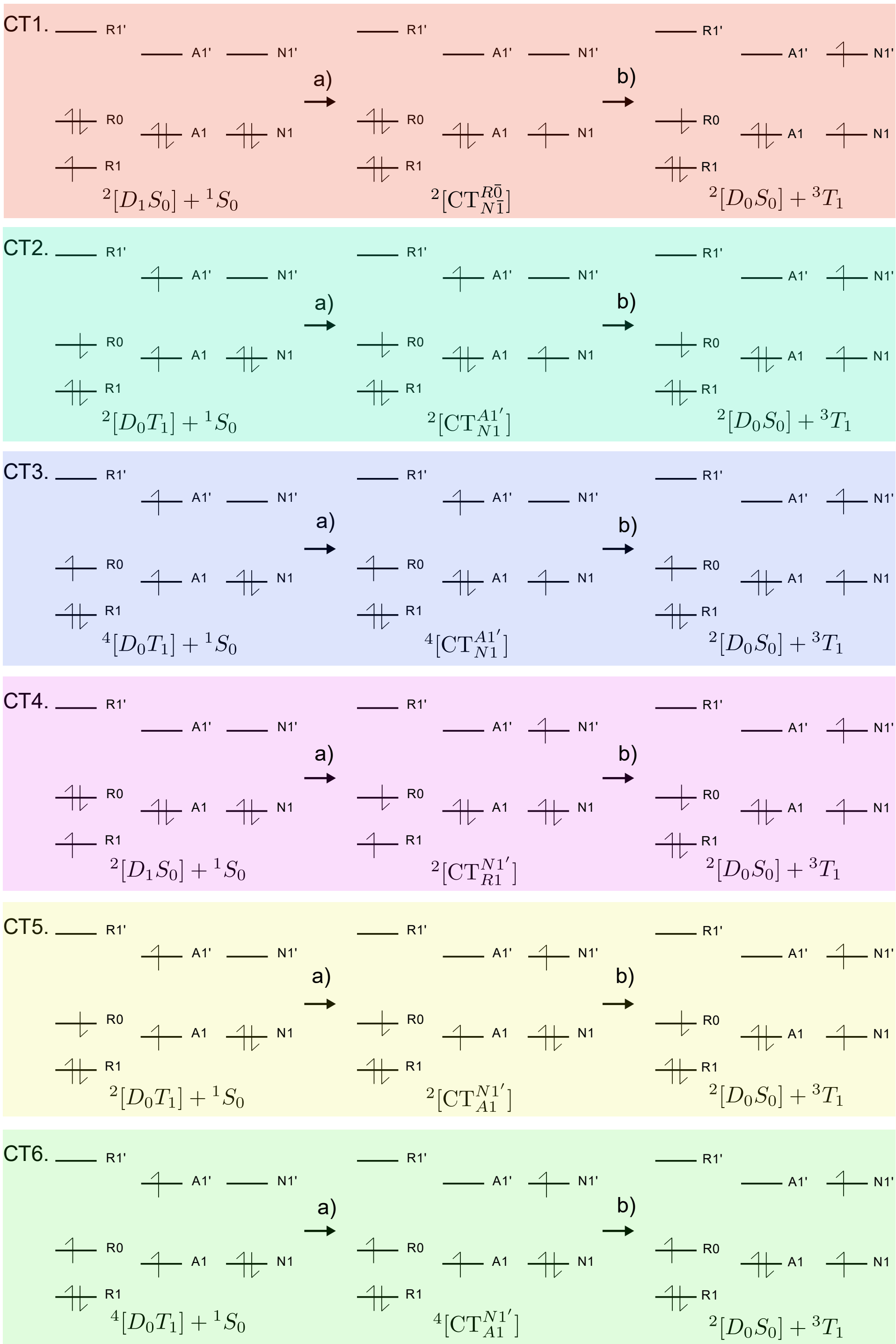

Figure S5. *Molecular orbital diagrams for charge-transfer mechanisms for energy transfer from radical-acene system to annihilator. 'R', 'A' and 'N' refer to orbitals on the radical, acene and annihilator respectively. Orbital 0 is the SOMO, 1 the HOMO and 1' the LUMO. Note that some states are spin-adapted by including multiple spin configurations, however, for simplicity only a single determinant is shown in each MO diagram. The following algebra was derived using the spin-adapted states. Further details can be found in SI ref. 2 and 4.*

Two-step mechanisms via charge-transfer states:

$$^2[D_1S_0] + {}^1[S_0] \rightarrow {}^2\left[\mathrm{CT}^{\overline{R0}}_{N1}\right] \rightarrow {}^2[D_0S_0] + {}^3[T_1] \qquad \text{S2.1}$$

$$^2[D_0T_1] + {}^1[S_0] \rightarrow {}^2\left[\mathrm{CT}^{A1'}_{N1}\right] \rightarrow {}^2[D_0S_0] + {}^3[T_1] \qquad \text{S2.2}$$

$$^4[D_0T_1] + {}^1[S_0] \rightarrow {}^4\left[\mathrm{CT}^{A1'}_{N1}\right] \rightarrow {}^2[D_0S_0] + {}^3[T_1] \qquad \text{S2.3}$$

$$^2[D_1S_0] + {}^1[S_0] \rightarrow {}^2\left[\mathrm{CT}^{N1'}_{R1}\right] \rightarrow {}^2[D_0S_0] + {}^3[T_1] \qquad \text{S2.4}$$

$$^2[D_0T_1] + {}^1[S_0] \rightarrow {}^2\left[\mathrm{CT}_{A1}^{N1'}\right] \rightarrow {}^2[D_0S_0] + {}^3[T_1] \quad \text{S2.5}$$
$$^4[D_0T_1] + {}^1[S_0] \rightarrow {}^4\left[\mathrm{CT}_{A1}^{N1'}\right] \rightarrow {}^2[D_0S_0] + {}^3[T_1] \quad \text{S2.6}$$

We have derived the free energy changes to forming the CT states in each of the six mechanisms, shown below in Equation S3.

Free energy change for charge-transfer mechanisms:

$$^2[D_1S_0] + {}^1[S_0] \rightarrow {}^2\left[\mathrm{CT}_{\overline{N1}}^{\overline{R0}}\right]: -\epsilon_{N1} + \epsilon_{R1} - J_{N1,R0} + J_{R1,R0} + \frac{1}{2}\left(K_{N1,R0} - K_{R1,R0}\right) \quad \text{S3.1}$$
$$^2[D_0T_1] + {}^1[S_0] \rightarrow {}^2\left[\mathrm{CT}_{N1}^{A1'}\right]: -\epsilon_{N1} + \epsilon_{A1} - J_{N1,A1'} + J_{A1,A1'} + K_{N1,R0} - K_{A1,R0} \quad \text{S3.2}$$
$$^4[D_0T_1] + {}^1[S_0] \rightarrow {}^4\left[\mathrm{CT}_{N1}^{A1'}\right]: -\epsilon_{N1} + \epsilon_{A1} - J_{N1,A1'} + J_{A1,A1'} - \frac{1}{2}\left(K_{N1,R0} - K_{A1,R0}\right) \quad \text{S3.3}$$
$$^2[D_1S_0] + {}^1[S_0] \rightarrow {}^2\left[\mathrm{CT}_{R1}^{N1'}\right]: \epsilon_{N1'} - \epsilon_{R0} - J_{R1,N1'} + J_{R1,R0} - \frac{1}{2}J_{R0,R0} + K_{N1',R0} + \frac{1}{2}K_{R1,R0} \quad \text{S3.4}$$
$$^2[D_0T_1] + {}^1[S_0] \rightarrow {}^2\left[\mathrm{CT}_{A1}^{N1'}\right]: \epsilon_{N1'} - \epsilon_{A1'} - J_{A1,N1'} + J_{A1,A1'} + K_{N1',R0} - K_{A1',R0} \quad \text{S3.5}$$
$$^4[D_0T_1] + {}^1[S_0] \rightarrow {}^4\left[\mathrm{CT}_{A1}^{N1'}\right]: \epsilon_{N1'} - \epsilon_{A1'} - J_{A1,N1'} + J_{A1,A1'} - \frac{1}{2}\left(K_{N1',R0} - K_{A1',R0}\right) \quad \text{S3.6}$$

Examining Equations S3.1-S3.6, we see that the free energy change to a first approximation depends on the relative energies of the annihilator HOMO, radical HOMO and acene HOMO for RA^-N^+ mechanisms and of the annihilator LUMO, radical SOMO and acene LUMO for RA^+N^- mechanisms. If the mechanism goes via $^{2/4}[D_0T_1]$ (mechanisms CT2, CT3, CT5 and CT6), the free energy change will likely be very small as the annihilator and acene HOMOs and the annihilator and acene LUMOs will be very close in energy as they are both DPA. This analysis assumes that the orbital energy differences dominate the free energy change where J and K terms are small due to weak orbital overlap and/or spatial separation of orbitals. Supposing instead that the mechanism goes directly from $^2[D_1S_0]$ to the CT state (not via $^{2/4}[D_0T_1]$), if the radical HOMO is lower in energy than the annihilator HOMO this favours the RA^-N^+ mechanism (CT1) and disfavours the opposite RA^+N^- mechanism (CT4). This is in fact the case for TTM-1Cz-DPA:DPA owing to an annihilator HOMO → radical SOMO inter CT state shown in Figure S9.

We also derived the relevant coupling terms between a) the initial state and CT state, and b) between the CT state and the state which corresponds to the radical and acene in their ground states and the annihilator in a triplet state $^2[D_0S_0] + {}^3[T_1]$.

Coupling terms for charge-transfer mechanisms:

$$^2[D_1S_0] + {}^1[S_0] \rightarrow {}^2\left[\mathrm{CT}_{\overline{N1}}^{\overline{R0}}\right]: -F_{R1,N1} - (R1N1 \mid R0R0) + \frac{1}{2}(R1R0 \mid R0N1) \quad \text{S4.1.a}$$
$$^2\left[\mathrm{CT}_{\overline{N1}}^{\overline{R0}}\right] \rightarrow {}^2[D_0S_0] + {}^3[T_1]: \frac{3}{\sqrt{6}}\left[F_{R0,N1'} + \frac{1}{2}(R0N1' \mid R0R0) - (R0N1' \mid N1N1)\right] \quad \text{S4.1.b}$$
$$^2[D_0T_1] + {}^1[S_0] \rightarrow {}^2\left[\mathrm{CT}_{N1}^{A1'}\right]: -F_{A1,N1} + (A1R0 \mid R0N1) - (A1N1 \mid A1'A1') \quad \text{S4.2.a}$$
$$^2\left[\mathrm{CT}_{N1}^{A1'}\right] \rightarrow {}^2[D_0S_0] + {}^3[T_1]: F_{A1'N1'} + (A1'R0 \mid R0N1') - (N1N1 \mid N1'A1') \quad \text{S4.2.b}$$
$$^4[D_0T_1] + {}^1[S_0] \rightarrow {}^4\left[\mathrm{CT}_{N1}^{A1'}\right]: -F_{A1,N1} - \frac{1}{2}(R0A1 \mid N1R0) - (A1N1 \mid A1'A1') \quad \text{S4.3.a}$$
$$^4\left[\mathrm{CT}_{N1}^{A1'}\right] \rightarrow {}^2[D_0S_0] + {}^3[T_1]: F_{A1',N1'} - \frac{1}{2}(A1'R0 \mid R0N1') - (N1N1 \mid N1'A1') \quad \text{S4.3.b}$$
$$^2[D_1S_0] + {}^1[S_0] \rightarrow {}^2\left[\mathrm{CT}_{R1}^{N1'}\right]: \frac{3}{\sqrt{6}}\left[F_{R0,N1'} + \frac{1}{2}(R0N1' \mid R0R0) - (R0N1' \mid R1R1)\right] \quad \text{S4.4.a}$$
$$^2\left[\mathrm{CT}_{R1}^{N1'}\right] \rightarrow {}^2[D_0S_0] + {}^3[T_1]: -F_{R1,N1} + (R1R0 \mid R0N1) - (R1N1 \mid N1'N1') \quad \text{S4.4.b}$$
$$^2[D_0T_1] + {}^1[S_0] \rightarrow {}^2\left[\mathrm{CT}_{A1}^{N1'}\right]: F_{A1'N1'} + (A1'R0 \mid R0N1') - (A1A1 \mid N1'A1') \quad \text{S4.5.a}$$
$$^2\left[\mathrm{CT}_{A1}^{N1'}\right] \rightarrow {}^2[D_0S_0] + {}^3[T_1]: -F_{A1,N1} + (A1R0 \mid R0N1) - (A1N1 \mid N1'N1') \quad \text{S4.5.b}$$

$$^4[D_0T_1] + {}^1[S_0] \rightarrow {}^4[\mathrm{CT}_{A1}^{N1'}]: F_{A1',N1'} - \frac{1}{2}(A1'R0 \mid R0N1') - (A1A1 \mid N1'A1') \qquad \mathrm{S4.6.a}$$

$$^4[\mathrm{CT}_{A1}^{N1'}] \rightarrow {}^2[D_0S_0] + {}^3[T_1]: -F_{A1,N1} - \frac{1}{2}(R0A1 \mid R0N1) - (A1N1 \mid N1'N1') \qquad \mathrm{S4.6.b}$$

On inspecting the coupling elements, those for the $^2[D_1S_0]$ state (mechanisms CT1 and CT4 in Equations S4.1.a-b and S4.4.a-b) involve one- and two-electron integrals of orbitals on separated parts of the system (the annihilator and radical are bridged by the acene but do not directly contact) therefore the couplings are likely to be very small, disfavouring these mechanisms. On the other hand, the coupling elements for the CT mechanisms via $^{2/4}[D_0T_1]$ (CT2, CT3, CT5 and CT6 in Equations S4.2.a-b, S4.3.a-b, S4.5.a-b, S4.6.a-b) are likely to be much larger as they involve one- and two-electron integrals of orbitals on neighbouring groups. Based on our analysis of the coupling terms for the Dexter mechanisms, and the free energy changes and coupling terms for the two-step CT mechanisms, we can expect from electronic structure theory that the mechanism most likely goes via the $^{2/4}[D_0T_1]$ state. We note again that this analysis assumes that the J and K terms are small, and was investigated further computationally in quantum chemical calculations.

Quantum chemical calculations

Computational methods

Doublet ground state geometries of TTM-1Cz-DPA and TTM-1Cz-DPA:DPA dimers were optimized in the framework of unrestricted density functional theory (DFT) using the ωB97X-D exchange-correlation functional and the 6-31g(d,p) basis set. Vertical transition energies were computed using time-dependent DFT within the Tamm-Dancoff approximation[12] (TDA-DFT) at the CAM-B3LYP/6-311g(d,p) level. Excited state analysis was performed with the TheoDORE program.[13] Electronic couplings between the relevant excited states were evaluated within the Fragment Excitation Difference (FED)[14] scheme.

It is important to note that the DFT-based calculations on open-shell systems, including spin doublet and quartet states, may be affected by spin contamination. The lack of exact spin symmetry in these computed states can particularly impact state properties, such oscillator strengths and interstate couplings (FED). To address this, we systematically report the $\langle S^2 \rangle$ values for all computed states in the tables below.

For TTM-1Cz-DPA, electronic couplings were also evaluated via a diabatisation procedure using the Boys[15] localisation scheme, where the adiabatic states were computed at the restricted active-space configuration interaction (RASCI)[16] method and the def2-SVP basis set. The RAS-CI calculation was carried out with one hole and one particle contributions and a DFT reference configuration (CAM-B3LYP/def2-svp), considering a RAS2 space with five electrons in five orbitals. This approach produces spin-pure states (no spin contamination) and was shown in a previous study to provide reliable results for a system of similar nature[10] and constitute a reference for validating the FED couplings. For the dimer of DPA units, an active space of four electrons in four orbitals was considered.

All calculations were done using Gaussian 16,[17] ORCA[18] and Q-Chem[19] program packages. The DrawMol[20] program was used for visualisation of the natural transition orbitals. Further details of the computational methods are given in the main text.

HTTM-1Cz-DPA excited states

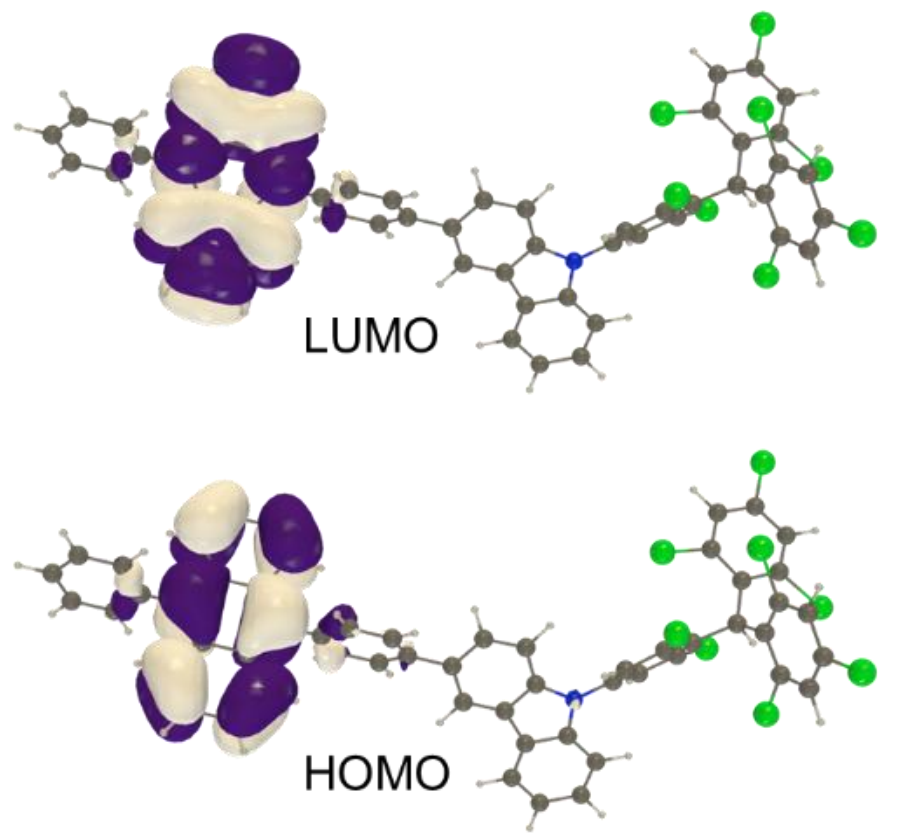

Figure S6. *HOMO and LUMO orbitals for HTTM-1Cz-DPA*

*Table **S1**. Vertical excitation energies and oscillator strengths of HTTM-1Cz-DPA computed at the CAM-B3LYP/6-311g(d,p) level.*

**state**	**E (eV)**	**f**	**HOMO → LUMO**
S_1	3.718	0.468	92%
T_1	2.144	0	90%

TTM-1Cz-DPA excited states

*Table **S2**. Vertical excitation energies and oscillator strengths of TTM-1Cz-DPA computed at the CAM-B3LYP/6-311g(d,p) level.*

**state**	**E (eV)**	**f**	**$\langle S^2 \rangle$**
D_0T_1	2.14	0	2.788
D_1S_0	2.85	0.113	1.073
D_2S_0	3.13	0.021	1.192
CT	3.54	0.005	0.959
D_0S_1	3.72	0.391	0.818

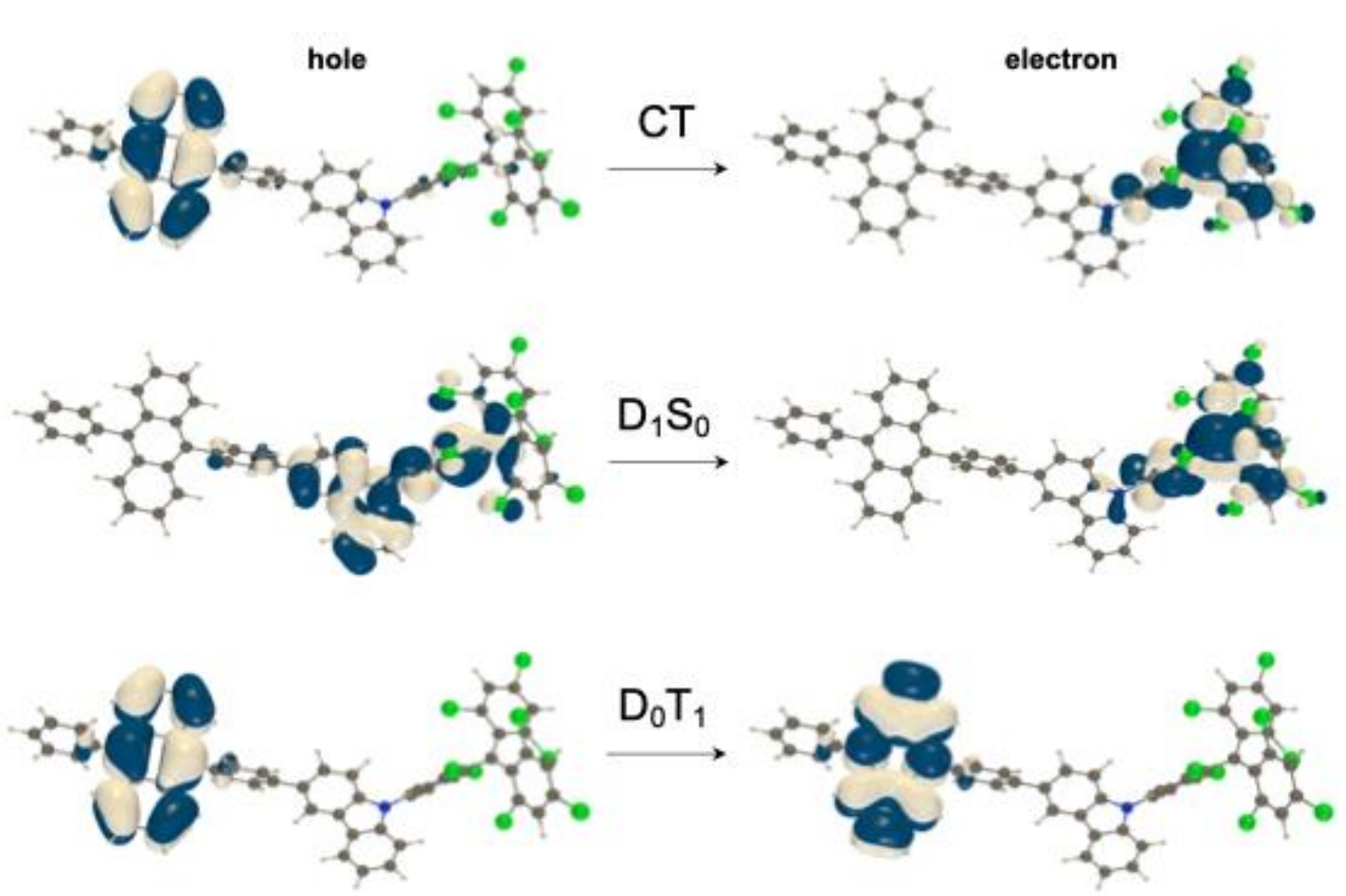

Figure S7. *Natural Transition Orbitals for the D_0T_1, D_1S_0 and CT states of TTM-1Cz-DPA.*

Table S3. *Electronic couplings (in meV) computed with the Fragment Excitation Difference (FED) method on the TDADFT excited states computed at the CAM-B3LYP/6-311g(d,p) level and through Boys diabatisation of the excited states computed at the RASCI/def2-SVP.*

coupled states	FED	RASCI
$\langle D_0T_1\|\hat{H}\|D_1S_0\rangle$	0.3	0.8
$\langle D_0T_1\|\hat{H}\|CT\rangle$	1.8	7.1
$\langle D_1S_0\|\hat{H}\|CT\rangle$	41.0	54.5

Notably, despite the spin contamination in TDDFT states, the FED couplings obtained are in good agreement with those from the Boys diabatisation of spin-pure doublet states calculated using RASCI. The calculations in Table S2 reveal the feasibility of a one-step Dexter-like doublet energy transfer from the bright $^2[D_1S_0]$ to $^2[D_0T_1]$ (Figure S15). Additionally, the relatively strong couplings of both states to the lowest intramolecular CT state, ^{2}[intra-CT], suggest the potential for a superexchange mechanism, with ^{2}[intra-CT] serving as a (virtual) mediator. This agrees with previous calculations performed on a TTM-1Cz-anthracene compound that was investigated for spin-optical interface applications.[10] We rule out two-step electron transfer mechanisms involving ^{2}[intra-CT], as our calculations indicated that this state is energetically too high.

TTM-1Cz-DPA:DPA dimers

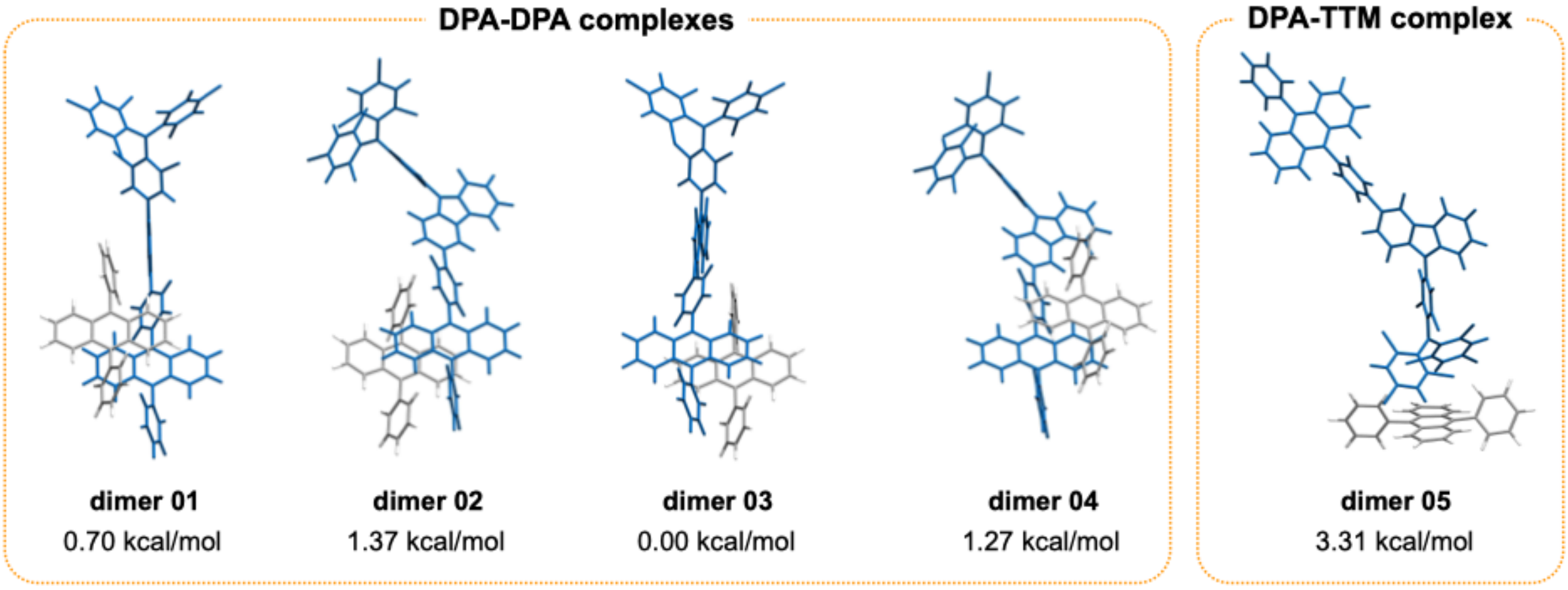

Figure S8. *Equilibrium structures of the five optimized TTM-1Cz-DPA:DPA dimers at the DFT level using ωB97X-D exchange-correlation functional and the 6-31g(d,p) basis set. DPA-DPA (referred as Rad-DPA:DPA complex in the SI text) and DPA-TTM (DPA:Rad-DPA complex in the SI text) complexes correspond to cases where the DPA and the TTM units of the TTM-1Cz-DPA sensitiser are in close proximity with the DPA annihilator.*

DPA:Rad-DPA complex

Table S4. *Vertical excitation energies and oscillator strengths of TTM-1Cz-DPA:DPA at its DPA-TTM complex equilibrium structure computed at the CAM-B3LYP/6-311g(d,p) level.*

state	E (ev)	f	$\langle S^2 \rangle$
T_1 (DPA)	2.10	0	2.785
D_0T_1	2.14	0	2.786
inter-CT	2.85	0.006	0.870
D_1S_0	2.90	0.113	1.092
D_2S_0	3.13	0.013	1.189
S_1 (DPA)	3.63	0.175	0.898
intra-CT	3.67	0.172	1.064
D_0S_1	3.72	0.351	0.827

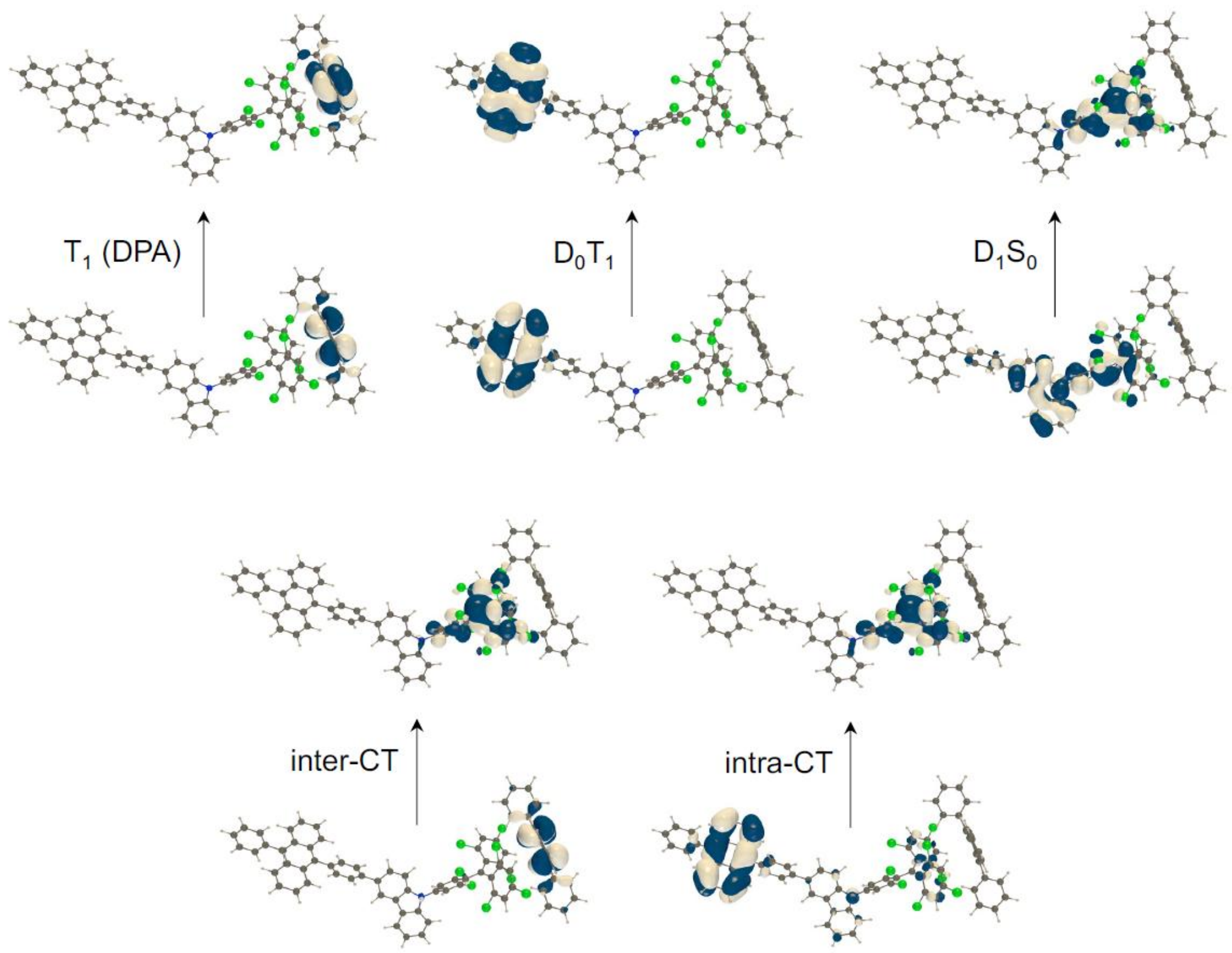

Figure S9. *Natural Transition Orbitals for the T_1(DPA), D_0T_1, D_1S_0 and (inter/intra) CT states of the TTM-1Cz-DPA:DPA dimer at its DPA-TTM complex geometry computed at the TDA-CAM-B3LYP/6-311g(d,p) level.*

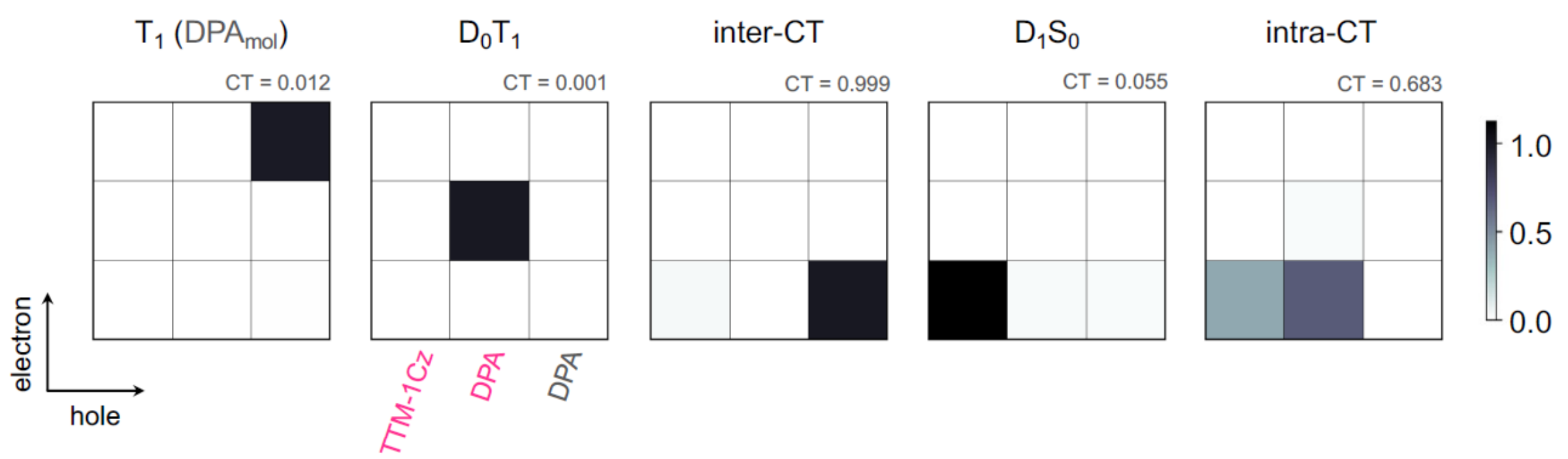

Figure S10. *Electron-hole correlation plots for the relevant excited states of the DPA:Rad-DPA complex computed at the TDA-CAM-B3LYP/6-311g(d,p) level considering three fragments: TTM-1Cz, DPA of the radical, and molecular DPA.*

Table S5. *FED electronic couplings (in meV) between the excited states of the DPA:Rad-DPA complex computed at the TDA-CAM-B3LYP/6-311g(d,p) level.*

coupled states	FED coupling
$\langle D_0T_1\|\hat{H}\|T_1(\text{DPA})\rangle$	0.0
$\langle D_0T_1\|\hat{H}\|D_1S_0\rangle$	0.3
$\langle D_0T_1\|\hat{H}\|\text{inter-CT}\rangle$	0.0
$\langle D_1S_0\|\hat{H}\|\text{inter-CT}\rangle$	7.1
$\langle D_0T_1\|\hat{H}\|\text{intra-CT}\rangle$	1.2
$\langle D_1S_0\|\hat{H}\|\text{intra-CT}\rangle$	11.8

Rad-DPA:DPA complex

Table S6. *Vertical excitation energies and oscillator strengths of Rad-DPA:DPA complex at its DPA-DPA complex equilibrium structure computed at the CAM-B3LYP/6-311g(d,p) level.*

state	nature	E (ev)	f	<S^2>
D_1	D_0T_1	2.06	0	2.787
D_2	T_1 (DPA)	2.09	0	2.787
D_3	D_1S_0	2.85	0.129	1.066
D_4	D_2S_0	3.13	0.021	1.192
D_5	CT_1	3.26	0.005	0.991
D_6	CT_2	3.37	0.009	1.299
D_{10}	CT_3	3.49	0.001	1.019

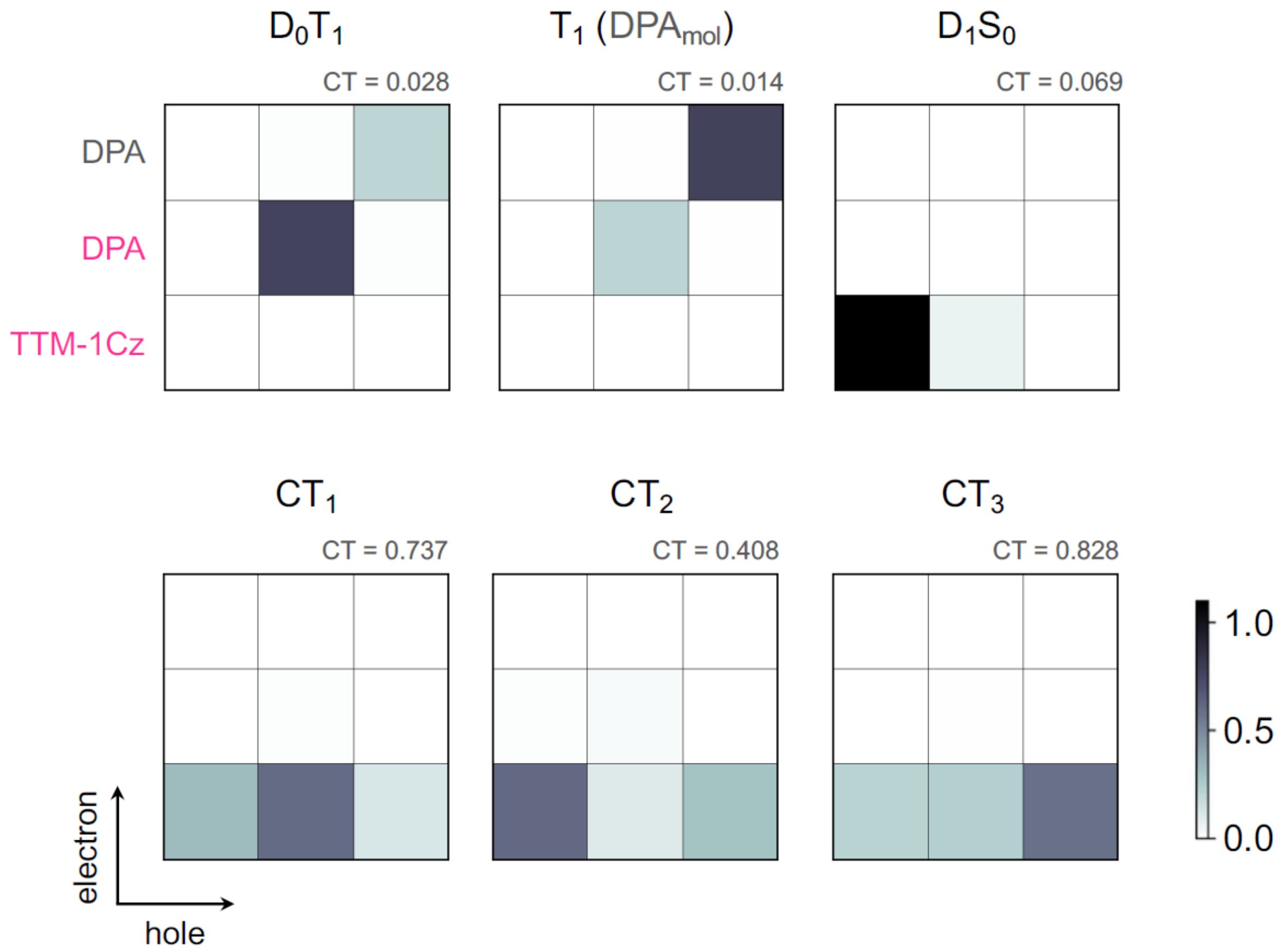

Figure S11. *Electron-hole correlation plots for the relevant excited states of the Rad-DPA:DPA complex computed at the TDA-CAM-B3LYP/6-311g(d,p) level considering three fragments: TTM-1Cz, DPA of the sensitizer, and molecular DPA.*

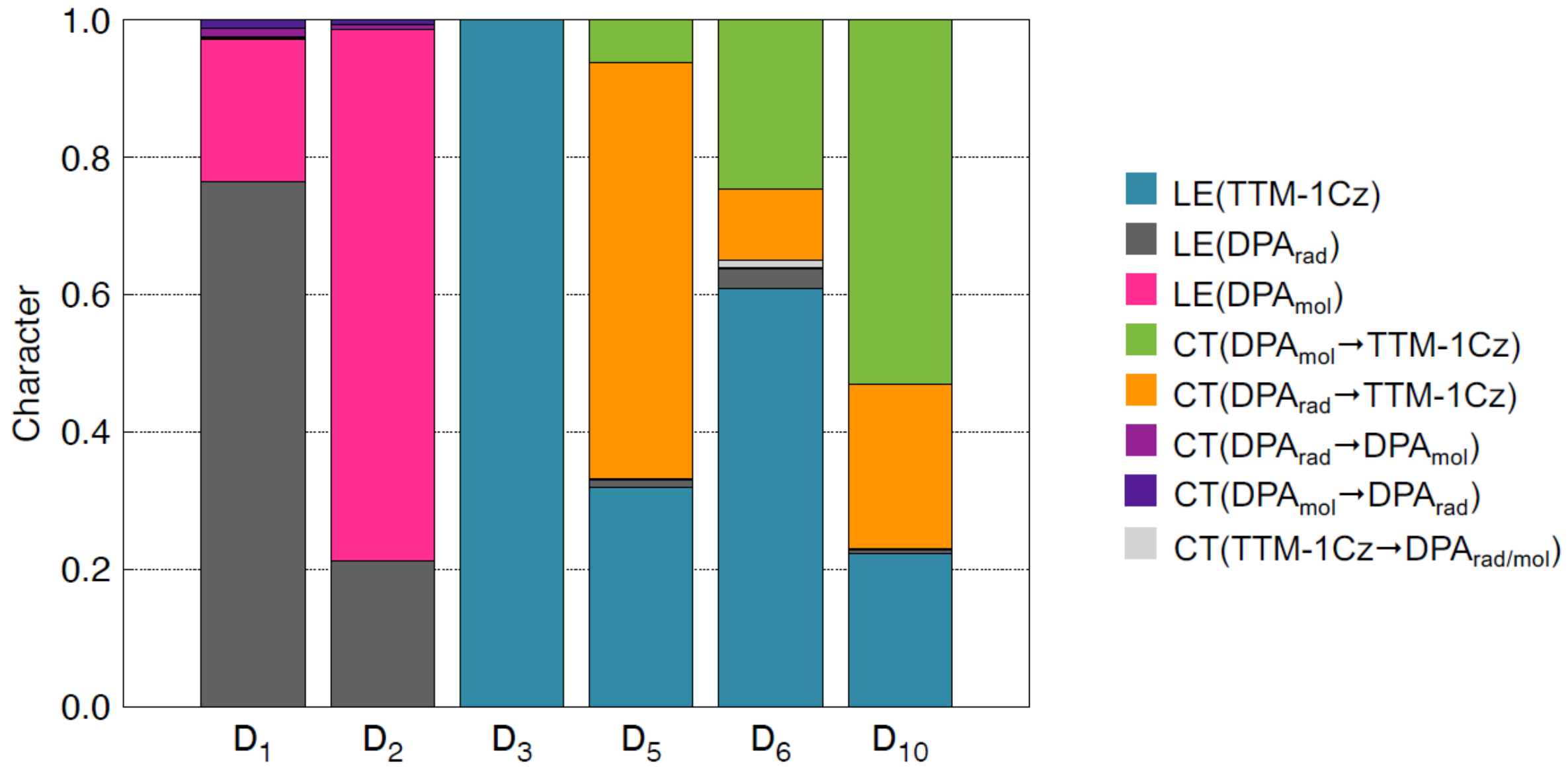

Figure S12. *Fragment-based analysis of the relevant doublet excited states computed at the TDA-CAM-B3LYP/6-311g(d,p) level of the Rad-DPA:DPA complex. The excited-state character (in %) is evaluated considering a partition of the system into three fragments: TTM-1Cz, DPA of the sensitizer, and molecular DPA.*

Table S7. *FED electronic couplings (in meV) between the excited states of the Rad-DPA:DPA complex computed at the TDA-CAM-B3LYP/6-311g(d,p) level. Two different fragmentation schemes, a and b, are considered (see footnote and Figure* ***S8****).*

coupled states	FED coupling
$\langle D_0T_1\|\hat{H}\|T_1(DPA)\rangle$	13.2[b]
$\langle D_0T_1\|\hat{H}\|D_1S_0\rangle$	2.5[a]
$\langle T_1(DPA)\|\hat{H}\|D_1S_0\rangle$	1.4[a]
$\langle D_0T_1\|\hat{H}\|CT_1\rangle$	7.6[a]
$\langle T_1(DPA)\|\hat{H}\|CT_1\rangle$	3.9[a]
$\langle D_1S_0\|\hat{H}\|CT_1\rangle$	91.1[a]
$\langle D_0T_1\|\hat{H}\|CT_2\rangle$	10.8[b]
$\langle T_1(DPA)\|\hat{H}\|CT_2\rangle$	4.6[b]
$\langle D_1S_0\|\hat{H}\|CT_2\rangle$	3.2[b]
$\langle D_0T_1\|\hat{H}\|CT_3\rangle$	4.4[b]
$\langle T_1(DPA)\|\hat{H}\|CT_3\rangle$	3.1[b]
$\langle D_1S_0\|\hat{H}\|CT_3\rangle$	2.7[b]

[a]FED fragments: TTM-1Cz and DPA:DPA dimer
[b]FED fragments: TTM-1Cz-DPA and DPA

FED fragmentation schemes

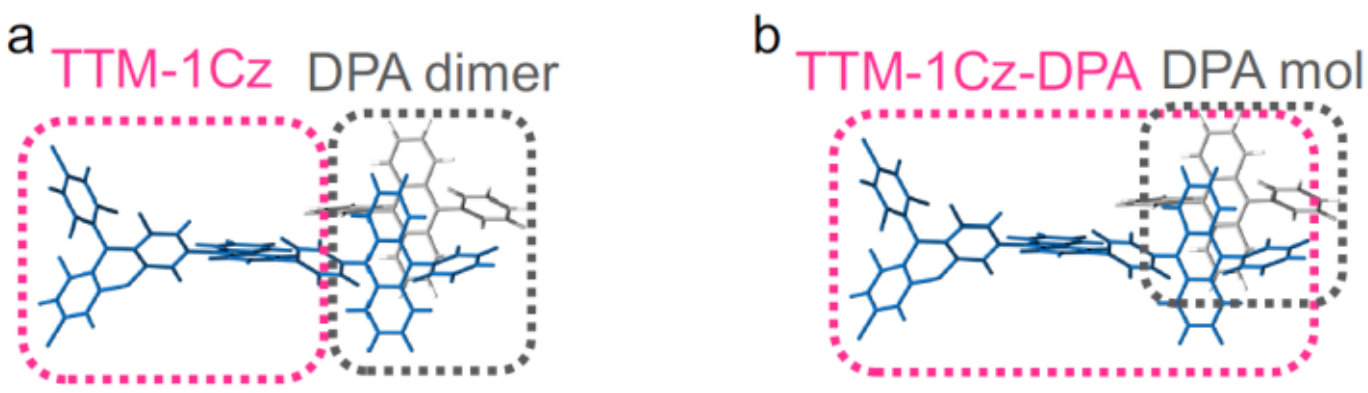

Figure S13: *Different fragmentation scheme used for the FED calculations: a. FED fragments: TTM-1Cz and DPA:DPA dimer and b. FED fragments: TTM-1Cz-DPA and DPA.*

These couplings should be interpreted with caution, as the mixing of multiple states with different electronic characters calls into question the validity of a two-state approach, such as FED, for calculating electronic couplings between diabatic states. This is particularly relevant for couplings involving states D_5, D_6, and D_{10}, where state analysis indicates that these excitations involve the mixing of at least three diabatic states (Figures S11 and S12).

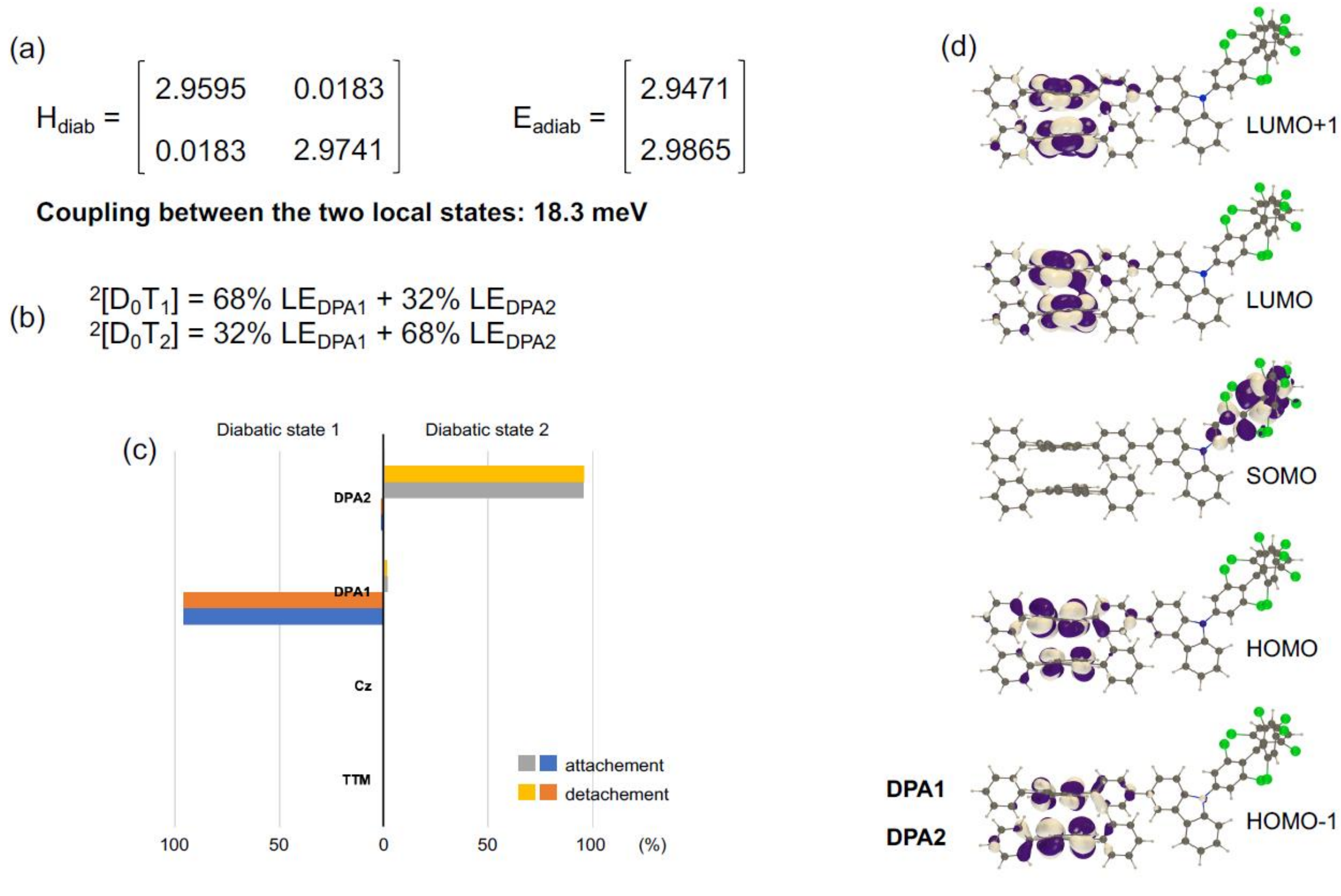

Figure S14: *Results of the Boys diabatisation of D_0T_1 and $T_1(DPA)$ as obtained with the RASCI method with one hole and one particle contributions considering an (5,5) active space: a) Diabatic Hamiltonian resulting from the Boys diabatiation and initial adiabatic energies; b) Decomposition of the two lower-lying RASCI adiabatic states in terms of the diabatic states; c) Nature of the diabatic states localized on the DPA unit (DPA1, 96% weight) of the sensitiser (state 1) and DPA annihilator (state 2, DPA2, 96% weight); d) Spatial localisation of the five orbitals included in the RASCI calculation.*

Proposed mechanisms for energy transfer

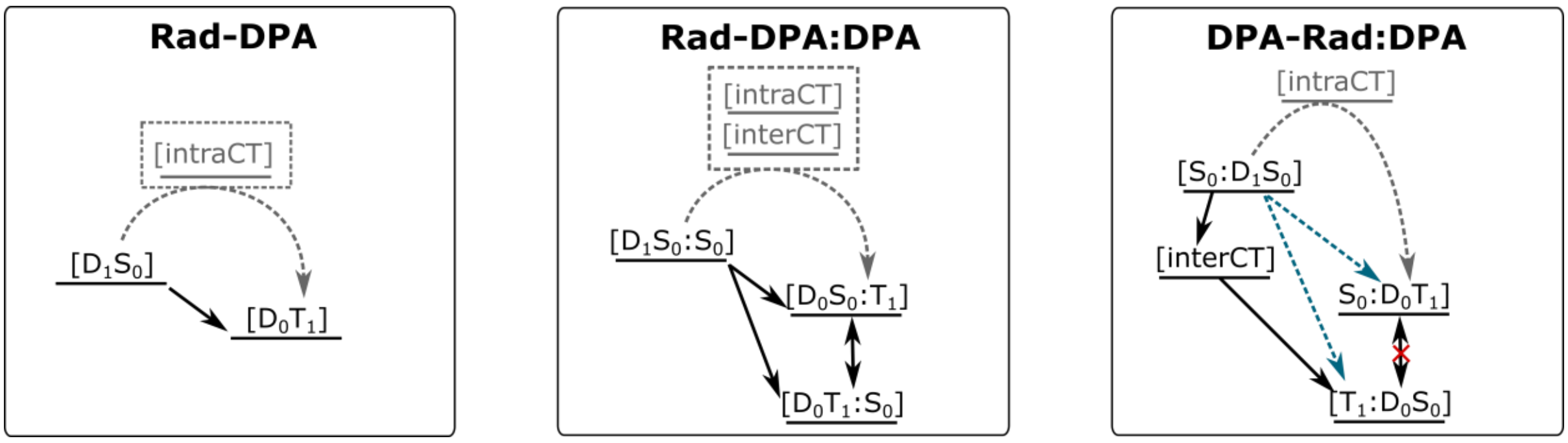

Figure S15. *State diagram representing the low-lying levels and energy transfer mechanisms from the excited D1 state of the TTM-1Cz (Rad) moiety in the Rad-DPA molecule (left), Rad-DPA:DPA (centre) and DPA-Rad:DPA (right) complexes. Dashed lines indicate mediated mechanisms. For clarity, spin multiplicities of electronic states are omitted. Rad-DPA and Rad-DPA:DPA are present in the main text but repeated here for clarity.*

We performed quantum chemical calculations on both the sensitiser alone (TTM-1Cz-DPA) and dimers of the sensitiser and DPA. Time-dependent density functional theory (TD-DFT) calculations revealed an electronic structure and intramolecular photophysics similar to the TTM-1Cz-anthracene compound previously characterised for spin-optical interface applications (Figure S15, 'Rad-DPA').[25] Namely, the doublet excited state of the TTM-1Cz radical $^2[D_1S_0]$ lies higher in energy with respect to the trip-doublet and trip-quartet $^{2,4}[D_0T_1]$ excited states. We also find an intramolecular charge transfer state, ^{2}[intra-CT], at higher energy where the hole is mostly localised on the DPA unit and the electron on the TTM core (Table S1). Our calculations suggest that transfer from $^2[D_1S_0]$ to $^2[D_0T_1]$ (Figure S15) is feasible by both one-step Dexter transfer or superexchange mechanism with ^{2}[intra-CT] as mediator.

Two key arrangements of TTM-1Cz-DPA and DPA were identified: 'Rad-DPA:DPA' where the DPA annihilator is in close contact with the DPA of the photosensitiser; and 'DPA:Rad-DPA' (Figure S15) where the DPA annihilator is positioned near the TTM radical. TD-DFT calculations showed that intermolecular formation of the triplet on the DPA (*i.e.* intermolecular trip-doublet $^2[D_0S_0:T_1]$, Figure S15 'Rad-DPA:DPA') was possible by direct Dexter transfer from the bright excited doublet, $^2[D_1S_0:S_0]$, or in a two-step process involving internal conversion to 'pre-formed triplets' from $^2[D_0T_1:S_0]$. Here trip-doublet formation mechanisms can occur by one-step Dexter energy transfer (see Table S6) or superexchange that is mediated by both inter- and intra-CT states. The possibility of short π-stacking arrangements between the two DPA units can also trigger strong exciton coupling between the two local trip-doublet states, about 13 meV at the TD-DFT (Table S6) and 18 meV at the RASCI level (Figure S13), facilitating efficient one-step Dexter triplet transfer between the sensitiser and annihilator DPA units. Intuitively, transfer using 'pre-formed' triplets between DPA groups in close-proximity for the Rad-DPA:DPA configuration is more favourable than DPA:Rad-DPA, although alternative mechanisms via intermolecular CT pathways could also enable efficient triplet sensitisation.

In the DPA:Rad-DPA complex, we identify two nearly degenerate low-lying states corresponding to the locally excited triplet states of the DPA units of the annihilator and sensitiser, $^2[T_1:D_0S_0]$ and $^2[S_0:D_0T_1]$ respectively (Figure S15 and Table S3). Here an intermolecular CT state ^{2}[inter-CT], with the DPA annihilator and the TTM moiety acting as electron donor and acceptor groups, respectively (Figure S9), was found at lower energy than the

bright doublet excited state localised on the TTM-1Cz fragment, $^2[S_0:D_1S_0]$. In this configuration, transfer from $^2[S_0:D_1S_0]$ to ^{2}[inter-CT] to $^2[T_1:D_0S_0]$ can be enabled by non-negligible electronic couplings between the states (Table S4). Interestingly, for the DPA:Rad-DPA configuration, it appears that the formation of the trip-doublet state on the sensitiser ('pre-formed' triplets) can be detrimental to the overall yield of annihilator triplets due to large spatial separation limiting triplet transfer.

4. Electron Spin Resonance (ESR)

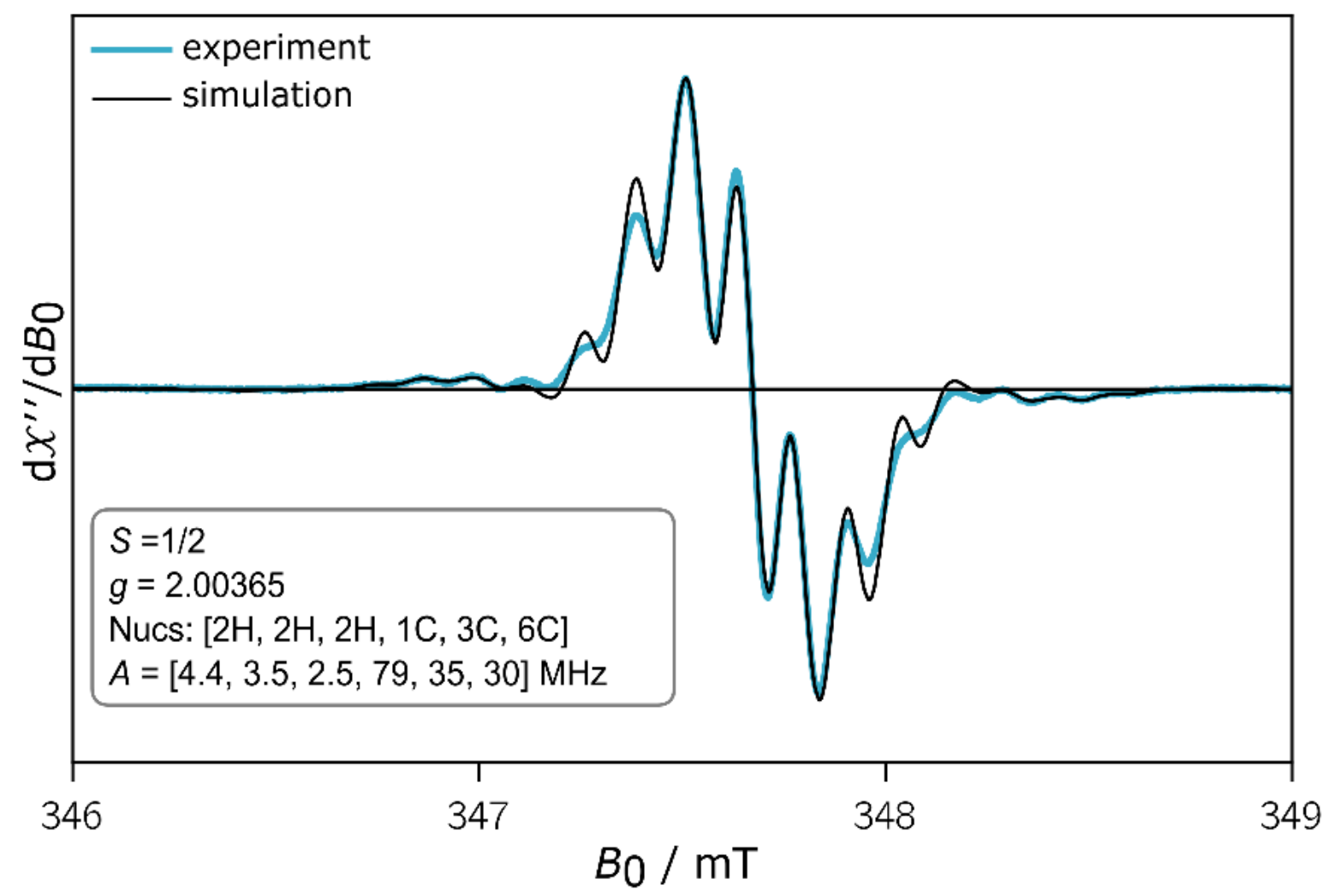

Figure S16. *Continuous wave ESR spectrum of TTM-1Cz-DPA (blue) with its EasySpin simulation (black) using the function garlic (EasySpin Version 6.0.4),[21,22] to determine the g-value and the hyperfine couplings A of the ground state radical. The simulation parameters are displayed in the figure.*

The trESR data were simulated using the function pepper of EasySpin Version 6.0.4.[21,22] The spin system is set up as a combination of two spins: One triplet spin and one doublet spin. The two spins are coupled using the exchange interaction parameter J and the axial and rhombic dipolar coupling *dip*. The g-value of the radical was taken from the fit of the dark state ESR spectrum, g-value and zero field splitting (ZFS) parameters D of the triplet are assumed to correspond to the values of anthracene. To match the multiplet polarisation, a linear combination of spin-polarised and purely absorptive triplet-doublet pair is used. The polarisation of the spectrum is the sum of the purely absorptive system and the spin polarised system. Following the law of parsimony, the purely absorptive triplet-doublet pair spectrum was modelled as a system with relative polarisations of the eigenstates corresponding to Boltzmann distribution, where the absolute polarisation/intensity has been scaled as described below. The initial populations of the spin polarised system are calculated from the zero-field (ZF) populations of the triplet, that are combined by direct product with the doublet populations. The initial polarisation of the coupled spin system in the 'uncoupled' basis is obtained. During fitting the g-values and the ZFS parameters were kept fixed. The initial populations of the spin-polarised system, the coupling parameters and the ratio of spin-polarised and purely absorptive populated system were varied. The final parameters are shown in table S1. The ratio of spin-polarised (no normalisation) and purely absorptive (normalised to maximum intensity) triplet-doublet pair was determined to 1:6.5. The sign of J cannot be determined from the simulation of the spectrum but was assumed to be positive (antiferromagnetic coupling). Only a lower bound but no exact value of J can be determined from the simulation

of the spectrum. The lower bound is about $J > 4000$ MHz, while the value given in table S1 is the one used for the final simulation.

Table S8. *Simulation parameters for the simulation of a triplet-doublet pair fitted to the experimental trESR spectrum of TTM-1Cz-DPA.*

$g_{Triplet}$	2.00521
$g_{Radical}$	2.00365
$D_{Triplet}$, $E_{Triplet}$ / MHz	2149, –250
$\|J\|$ / MHz	25000
dip / MHz	22, 60
ZF population triplet (ascending energy)	0.294, 0.261, 0.273
Population doublet states (ascending energy)	0.470, 0.478

5. Stern Volmer (Transient PL)

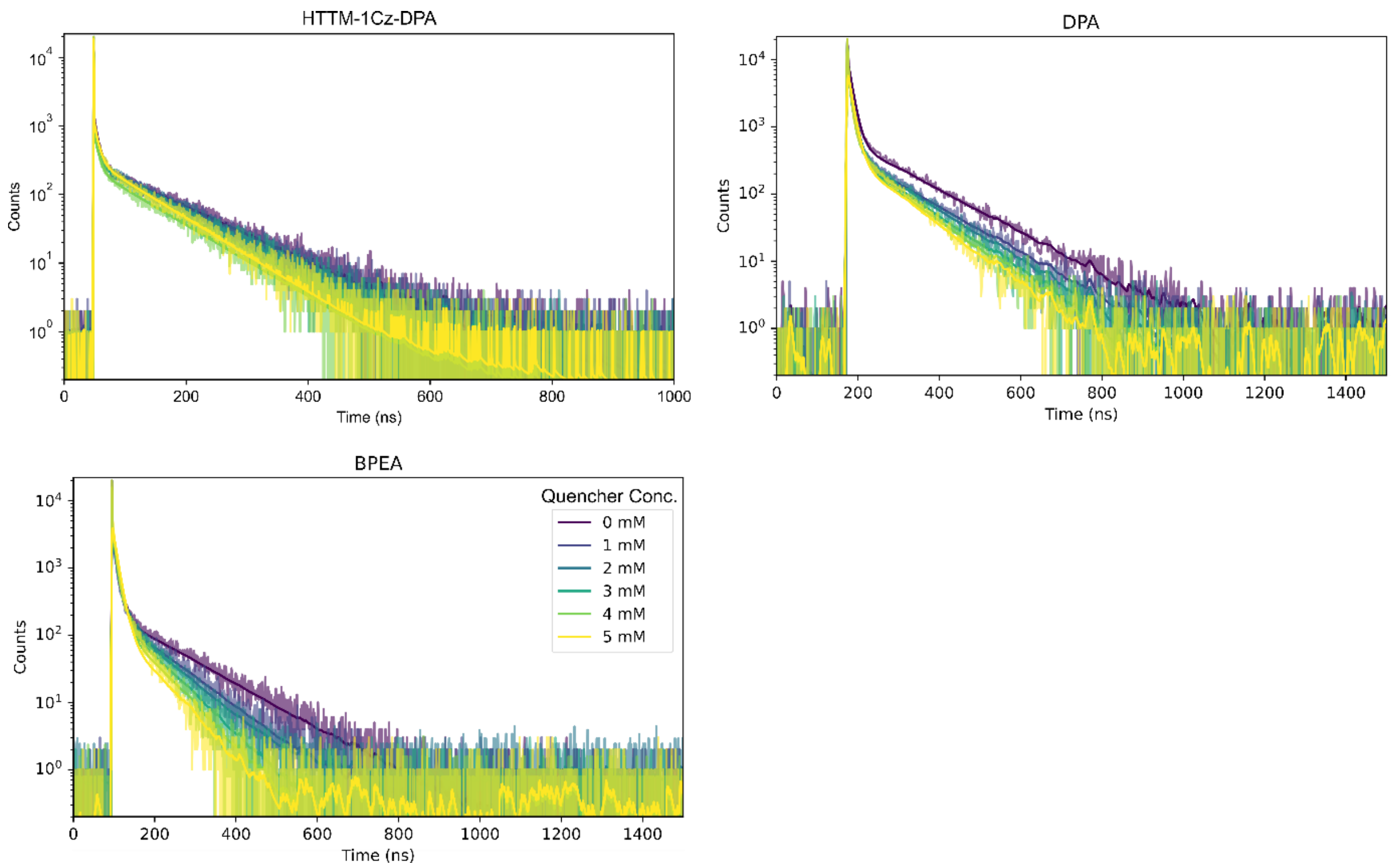

Figure S17. *Transient PL (TRPL) kinetics of the 730 nm emission from TTM-1Cz-DPA following 375 nm excitation, with and without HTTM-1Cz-DPA, DPA or BPEA quencher at different concentrations (1 — 5 mM). The darker lines show fitting to a biexponential decay. The second decay constant (τ_2) is used in the Stern-Volmer plot (main text, Figure* ***3e****).*

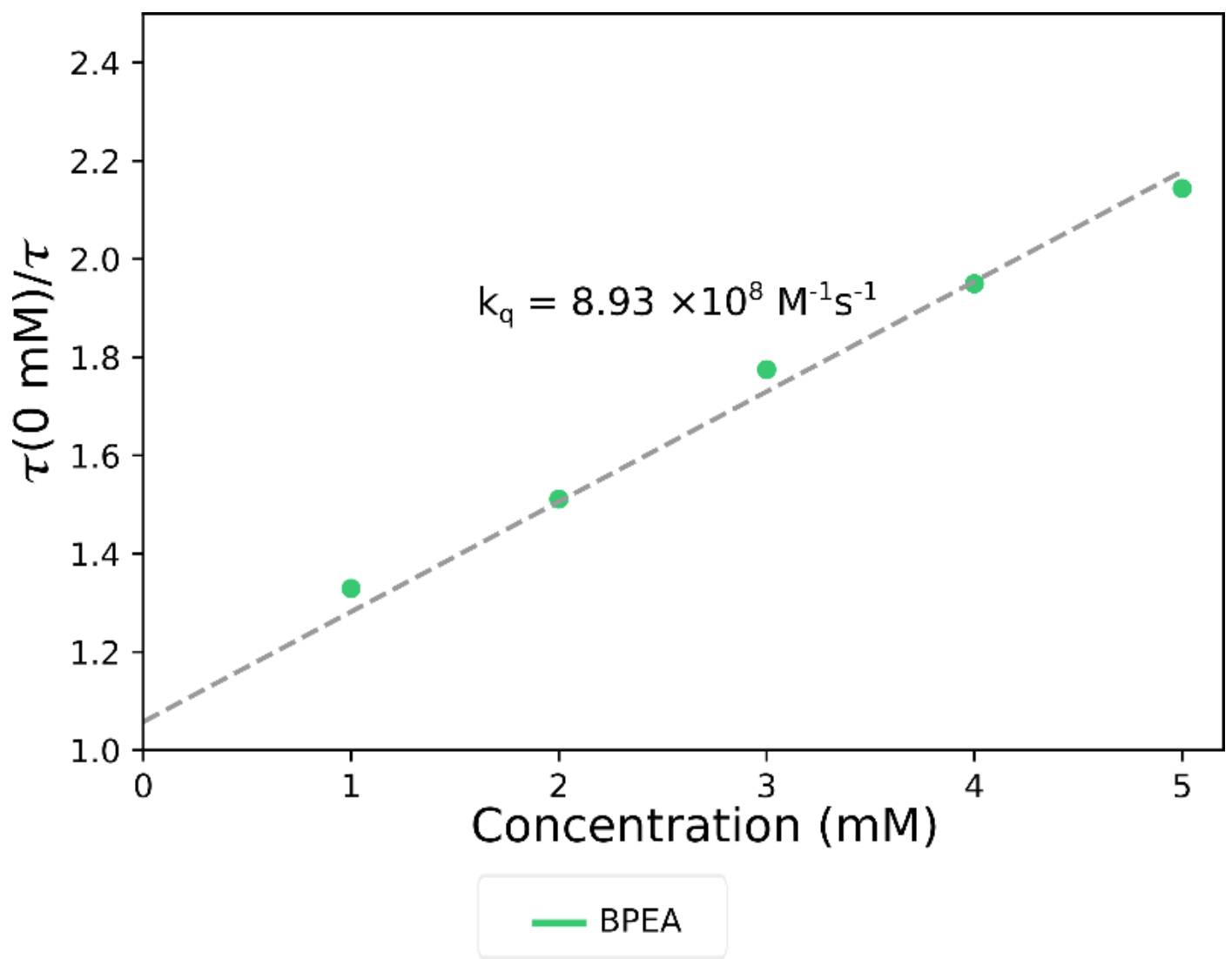

Figure S18. *Stern-Volmer plot for TTM-1Cz-DPA with BPEA using TRPL data shown in Figure* ***S17.***

6. BPEA power dependence

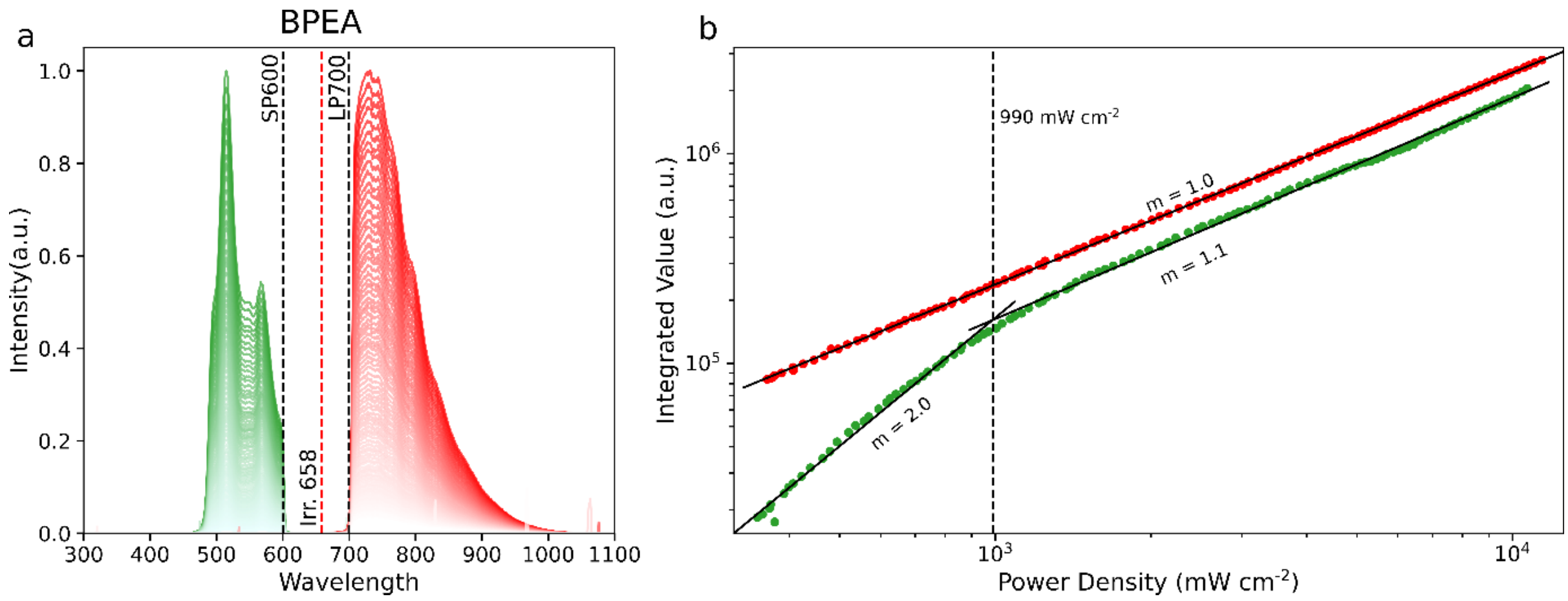

Figure S19. a *Normalised emission spectra for TTM-1Cz-DPA (50 µM) and BPEA (8 mM) in toluene, showing TTM-1Cz-DPA emission (red, 700 nm long-pass filter) and upconverted BPEA emission (green lines, 600 nm short-pass filter).* ***b*** *Power density dependence of TTM-1Cz-DPA emission (red markers) and upconverted emission of TTM-1Cz-DPA with BPEA (green markers) as the annihilator. The gradient of a linear fit, m, is given for both emission ranges.*

7. Stability study

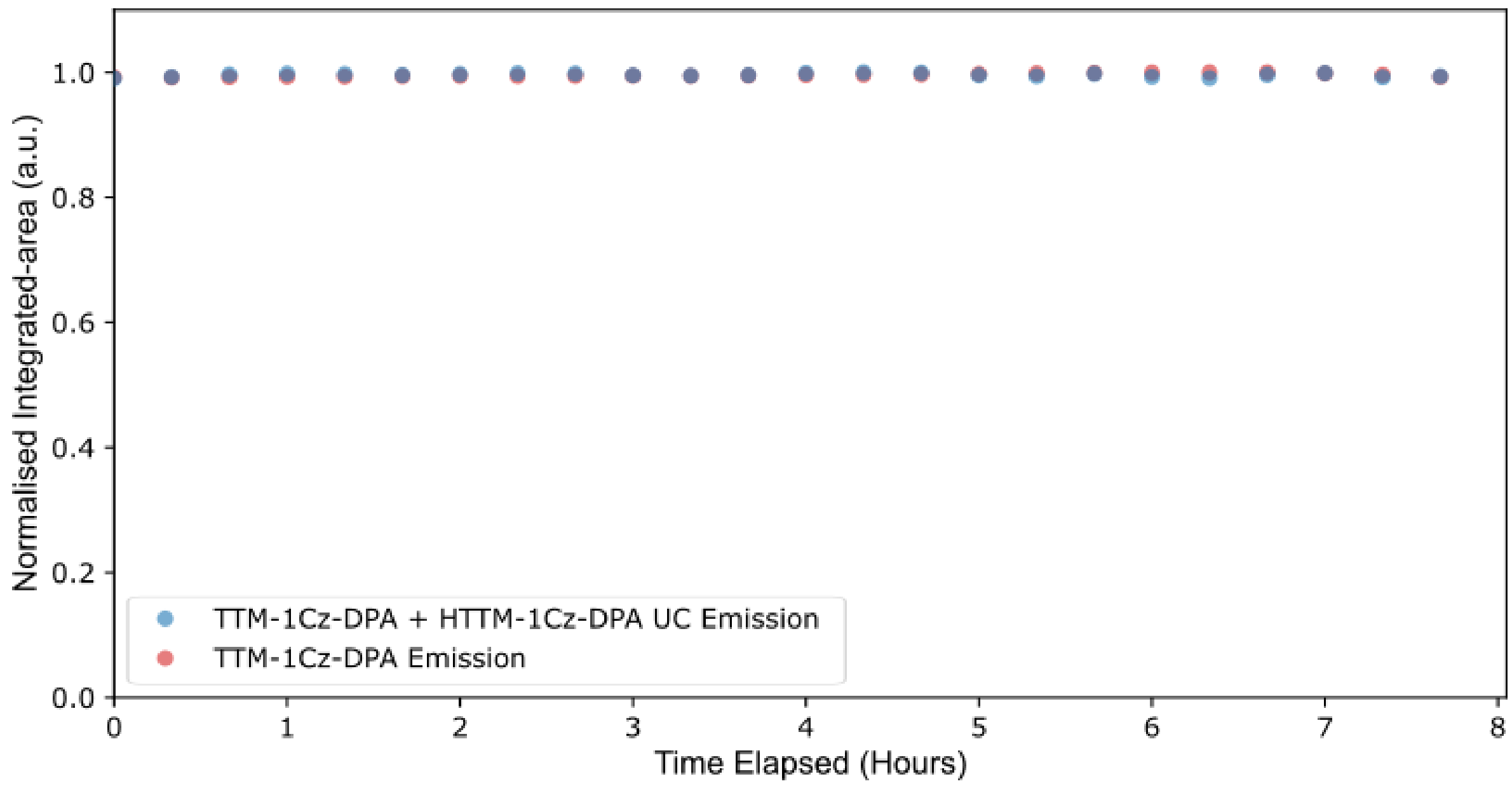

Figure S20. *Degradation study showing measurements over 8 hours for TTM-1Cz-DPA (50 µM) emission (700-800 nm) and upconverted emission (400-600 nm) from TTM-1Cz-DPA (50 µM) + HTTM-1Cz-DPA (10 mM) in deoxygenated toluene, excited at 661 nm (6 W cm^{-2}). Points are shown to overlap at near 100% for the duration of the experiment.*

8. Upconversion in films

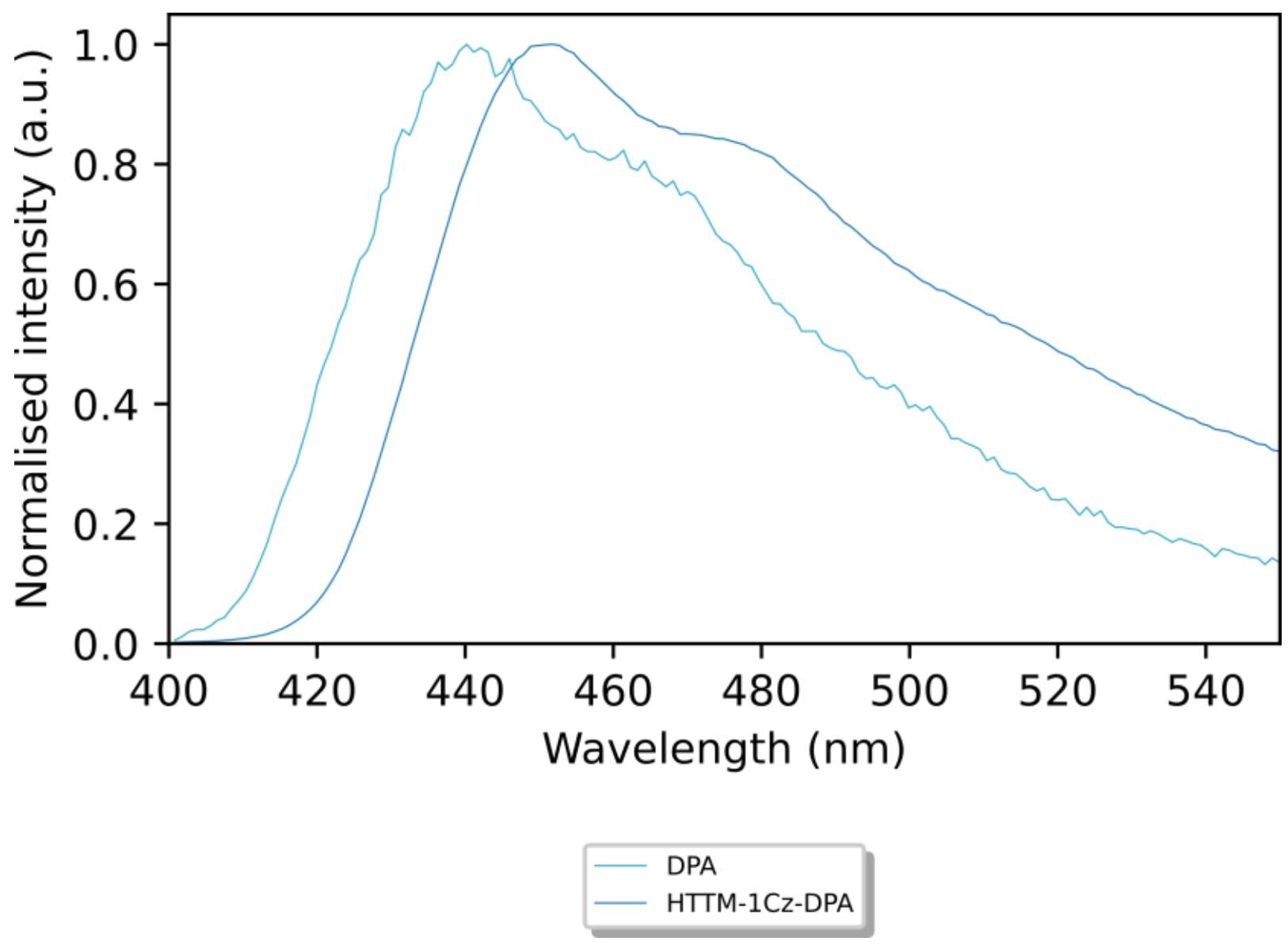

Figure S21. *Normalised upconverted emission in solid-state films for TTM-1Cz-DPA (sensitiser) with DPA (annihilator) or HTTM-1Cz-DPA (annihilator) in 1:200 ratio in PMMA. The films were dropcast from 200 mg ml^{-1} PMMA in chloroform using [sensitiser] = 100 µM and [annihilator] = 20 mM. Samples were irradiated at 661 nm (61 W cm^{-2}). Φ_{UC} = 0.001% for upconversion emission peak = 451 nm (energy shift = 0.87 eV) with HTTM-1Cz-DPA. Φ_{UC} = 0.0002% for upconversion emission peak = 440 nm (energy shift = 0.94 eV) with DPA.*

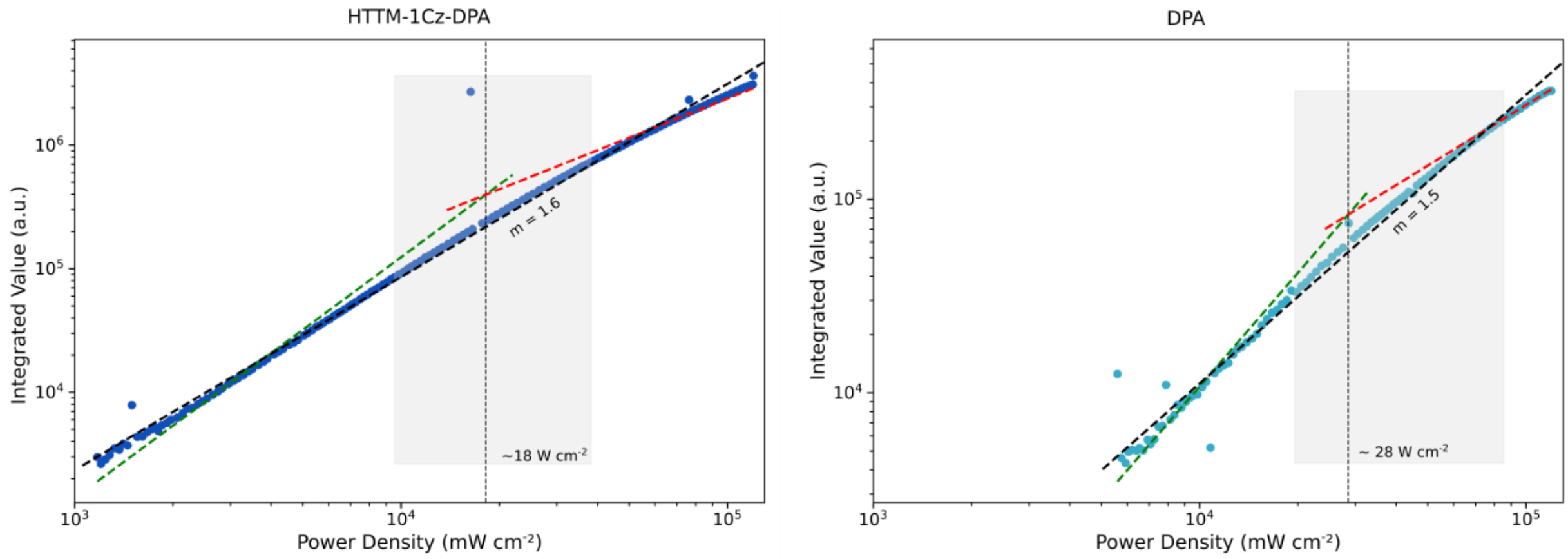

Figure S22. *Power density dependence on upconverted emission from solid-state films of TTM-1Cz-DPA (sensitiser) with DPA (annihilator) or HTTM-1Cz-DPA (annihilator) in 1:200 ratio in PMMA. The films were dropcast from 200 mg ml^{-1} PMMA in chloroform using [sensitiser] = 100 µM and [annihilator] = 20 mM. Samples were irradiated at 661 nm. Datapoints (grey region) were excluded to fit representative '2/1' gradients.*

9. HTTM-1Cz-DPA fraction

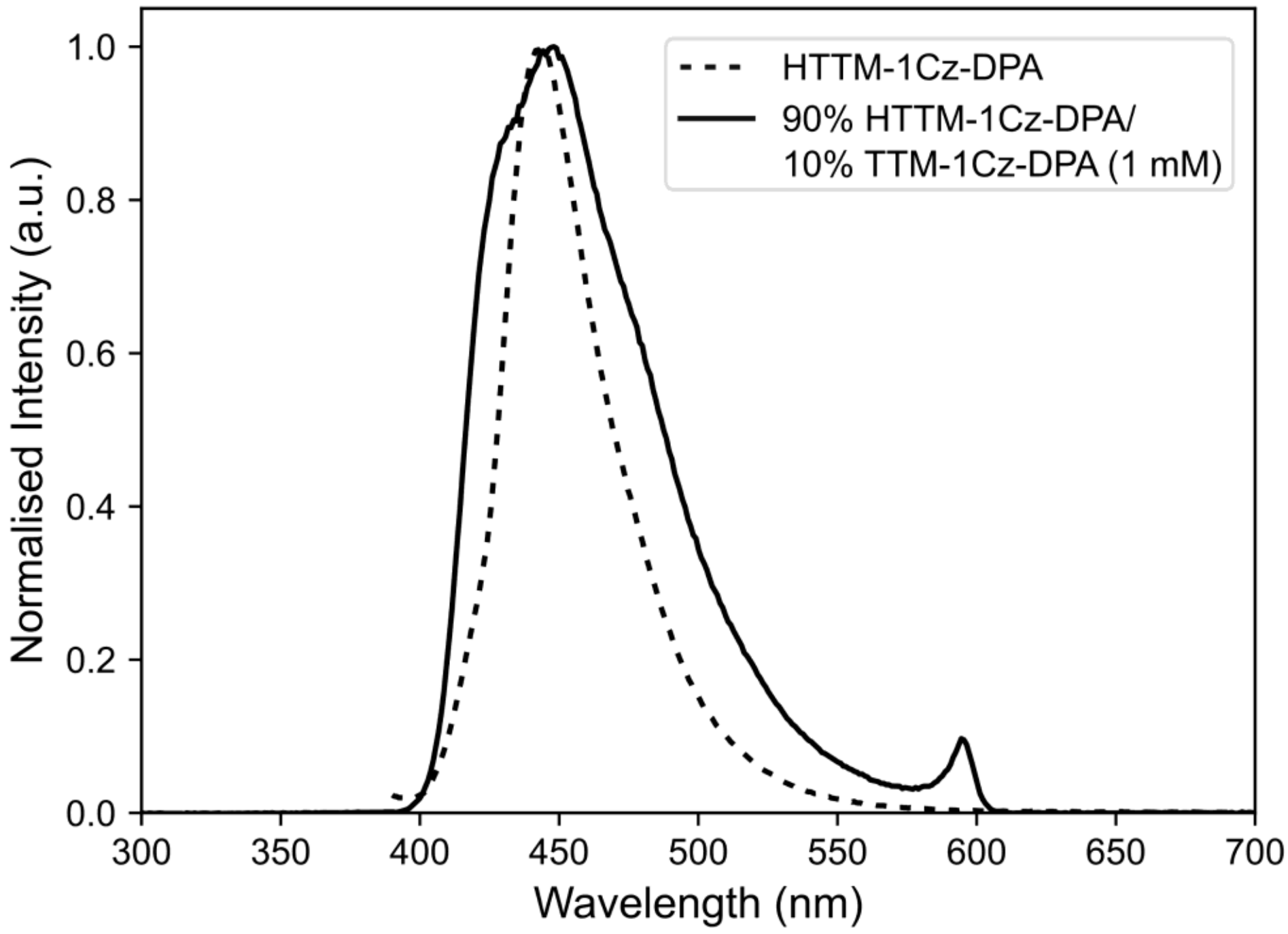

Figure S23. *Upconverted emission upon irradiation of 1mM 90% HTTM-1Cz-DPA / 10% TTM-1Cz-DPA with 661 nm (62 W cm^{-2}) laser (solid line). Emission spectra of HTTM-1Cz-DPA is given for reference (360 nm exc.) measured on a different instrument.*

Table S9. *Overview showing whether upconversion was observed for HTTM-1Cz-DPA/TTM-1Cz-DPA mixtures in toluene, irradiated with 661 nm (62 Wcm^{-2}) laser.*

Total Concentration	HTTM-1Cz-DPA (%)	Upconversion Observed? (PLQE %)
10 mM	0	✕
	1	✕
	10	✕
	50	✓ (~0.0002)
	90	✓ (~ 0.0002)
1 mM	0	✕
	1	✕
	10	✕
	50	✕
	90	✓ (0.0173)
50 µM	0	✕
	1	✕
	10	✕
	50	✕
	90	✕

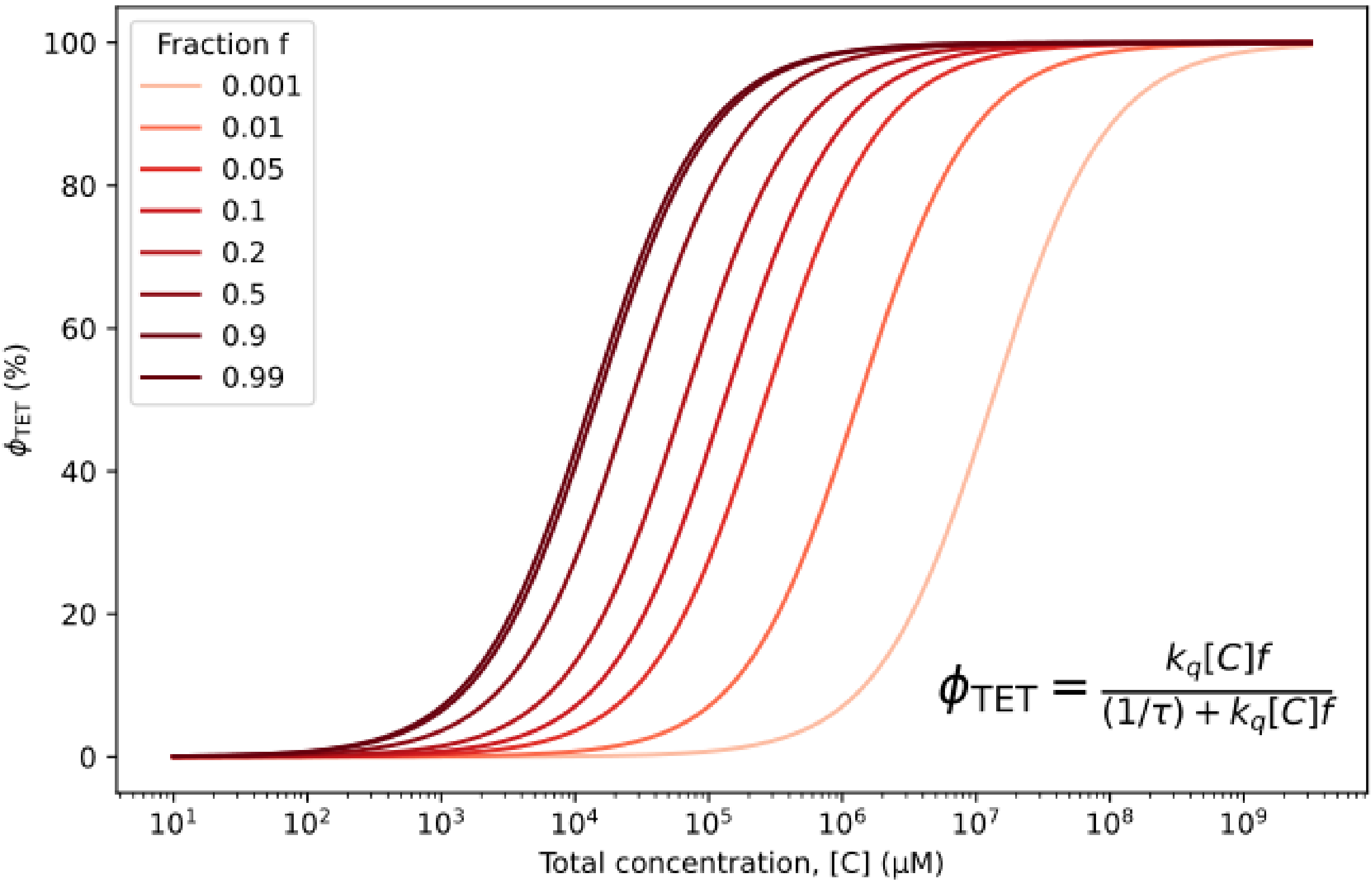

Figure S24. *Model showing effect on triplet energy transfer yield ϕ_{TET} with increasing total concentration [C] of radical-annihilator mixture and fraction f of hydrogenated precursor. From toluene solution, τ = 266 ns for excited-state lifetime of TTM-1Cz-DPA only; k_q = 2.85 ×10^8 $M^{-1}s^{-1}$ as determined from TTM-1Cz-DPA intermolecular energy transfer rate constant with HTTM-1Cz-DPA.*

References:

(1) Peng, Q.; Obolda, A.; Zhang, M.; Li, F. Organic Light-Emitting Diodes Using a Neutral π Radical as Emitter: The Emission from a Doublet. *Angew. Chem. Int. Ed.* **2015**, *54* (24), 7091–7095. https://doi.org/10.1002/anie.201500242.

(2) Hele, T. J. H. On the Electronic Structure of Alternant Conjugated Organic Radicals for Light-Emitting Diode Applications. In *Physical Chemistry of Semiconductor Materials and Interfaces XX*; SPIE, 2021; Vol. 11799, pp 57–73. https://doi.org/10.1117/12.2593712.

(3) Green, J. D.; Hele, T. J. H. ExROPPP: Fast, Accurate, and Spin-Pure Calculation of the Electronically Excited States of Organic Hydrocarbon Radicals. *J. Chem. Phys.* **2024**, *160* (16), 164110. https://doi.org/10.1063/5.0191373.

(4) Green, J. D.; Fuemmeler, E. G.; Hele, T. J. H. Inverse Molecular Design from First Principles: Tailoring Organic Chromophore Spectra for Optoelectronic Applications. *J. Chem. Phys.* **2022**, *156* (18), 180901. https://doi.org/10.1063/5.0082311.

(5) Ai, X.; Evans, E. W.; Dong, S.; Gillett, A. J.; Guo, H.; Chen, Y.; Hele, T. J. H.; Friend, R. H.; Li, F. Efficient Radical-Based Light-Emitting Diodes with Doublet Emission. *Nature* **2018**, *563* (7732), 536–540. https://doi.org/10.1038/s41586-018-0695-9.

(6) Abdurahman, A.; Hele, T. J. H.; Gu, Q.; Zhang, J.; Peng, Q.; Zhang, M.; Friend, R. H.; Li, F.; Evans, E. W. Understanding the Luminescent Nature of Organic Radicals for Efficient Doublet Emitters and Pure-Red Light-Emitting Diodes. *Nat. Mater.* **2020**, *19* (11), 1224–1229. https://doi.org/10.1038/s41563-020-0705-9.

(7) Li, F.; Gillett, A. J.; Gu, Q.; Ding, J.; Chen, Z.; Hele, T. J. H.; Myers, W. K.; Friend, R. H.; Evans, E. W. Singlet and Triplet to Doublet Energy Transfer: Improving Organic

Light-Emitting Diodes with Radicals. *Nat. Commun.* **2022**, *13* (1), 2744. https://doi.org/10.1038/s41467-022-29759-7.
(8) Hudson, J. M.; Hele, T. J. H.; Evans, E. W. Efficient Light-Emitting Diodes from Organic Radicals with Doublet Emission. *J. Appl. Phys.* **2021**, *129* (18), 180901. https://doi.org/10.1063/5.0047636.
(9) Hele, T. J. H.; Fuemmeler, E. G.; Sanders, S. N.; Kumarasamy, E.; Sfeir, M. Y.; Campos, L. M.; Ananth, N. Anticipating Acene-Based Chromophore Spectra with Molecular Orbital Arguments. *J. Phys. Chem. A* **2019**, *123* (13), 2527–2536. https://doi.org/10.1021/acs.jpca.8b12222.
(10) Gorgon, S.; Lv, K.; Grüne, J.; Drummond, B. H.; Myers, W. K.; Londi, G.; Ricci, G.; Valverde, D.; Tonnelé, C.; Murto, P.; Romanov, A. S.; Casanova, D.; Dyakonov, V.; Sperlich, A.; Beljonne, D.; Olivier, Y.; Li, F.; Friend, R. H.; Evans, E. W. Reversible Spin-Optical Interface in Luminescent Organic Radicals. *Nature* **2023**, *620* (7974), 538–544. https://doi.org/10.1038/s41586-023-06222-1.
(11) Coulson, C. A.; Rushbrooke, G. S. Note on the Method of Molecular Orbitals. *Math. Proc. Camb. Philos. Soc.* **1940**, *36* (2), 193–200. https://doi.org/10.1017/S0305004100017163.
(12) Hirata, S.; Head-Gordon, M. Time-Dependent Density Functional Theory within the Tamm–Dancoff Approximation. *Chem. Phys. Lett.* **1999**, *314* (3), 291–299. https://doi.org/10.1016/S0009-2614(99)01149-5.
(13) Plasser, F. TheoDORE: A Toolbox for a Detailed and Automated Analysis of Electronic Excited State Computations. *J. Chem. Phys.* **2020**, *152* (8), 084108. https://doi.org/10.1063/1.5143076.
(14) Hsu, C.-P.; You, Z.-Q.; Chen, H.-C. Characterization of the Short-Range Couplings in Excitation Energy Transfer. *J. Phys. Chem. C* **2008**, *112* (4), 1204–1212. https://doi.org/10.1021/jp076512i.
(15) Subotnik, J. E.; Vura-Weis, J.; Sodt, A. J.; Ratner, M. A. Predicting Accurate Electronic Excitation Transfer Rates via Marcus Theory with Boys or Edmiston−Ruedenberg Localized Diabatization. *J. Phys. Chem. A* **2010**, *114* (33), 8665–8675. https://doi.org/10.1021/jp101235a.
(16) Casanova, D.; Head-Gordon, M. Restricted Active Space Spin-Flip Configuration Interaction Approach: Theory, Implementation and Examples. *Phys. Chem. Chem. Phys.* **2009**, *11* (42), 9779–9790. https://doi.org/10.1039/B911513G.
(17) Frisch, M. J.; Trucks, G. W.; Schlegel, H. B.; Scuseria, G. E.; Robb, M. A.; Cheeseman, J. R.; Scalmani, G.; Barone, V.; Petersson, G. A.; Nakatsuji, H.; Li, X.; Caricato, M.; Marenich, A. V.; Bloino, J.; Janesko, B. G.; Gomperts, R.; Mennucci, B.; Hratchian, H. P.; Ortiz, J. V.; Izmaylov, A. F.; Sonnenberg, J. L.; Williams-Young, D.; Ding, F.; Lipparini, F.; Egidi, F.; Goings, J.; Peng, B.; Petrone, A.; Henderson, T.; Ranasinghe, D.; Zakrzewski, V. G.; Gao, J.; Rega, N.; Zheng, G.; Liang, W.; Hada, M.; Ehara, M.; Toyota, K.; Fukuda, R.; Hasegawa, J.; Ishida, M.; Nakajima, T.; Honda, Y.; Kitao, O.; Nakai, H.; Vreven, T.; Throssell, K.; Montgomery, J. A., Jr.; Peralta, J. E.; Ogliaro, F.; Bearpark, M. J.; Heyd, J. J.; Brothers, E. N.; Kudin, K. N.; Staroverov, V. N.; Keith, T. A.; Kobayashi, R.; Normand, J.; Raghavachari, K.; Rendell, A. P.; Burant, J. C.; Iyengar, S. S.; Tomasi, J.; Cossi, M.; Millam, J. M.; Klene, M.; Adamo, C.; Cammi, R.; Ochterski, J. W.; Martin, R. L.; Morokuma, K.; Farkas, O.; Foresman, J. B.; Fox, D. J. *Gaussian 16*, B.01; Wallingford, CT, 2016.
(18) Neese, F. Software Update: The ORCA Program System—Version 5.0. *WIREs Comput. Mol. Sci.* **2022**, *12* (5), e1606. https://doi.org/10.1002/wcms.1606.
(19) Epifanovsky, E.; Gilbert, A. T. B.; Feng, X.; Lee, J.; Mao, Y.; Mardirossian, N.; Pokhilko, P.; White, A. F.; Coons, M. P.; Dempwolff, A. L.; Gan, Z.; Hait, D.; Horn, P. R.; Jacobson, L. D.; Kaliman, I.; Kussmann, J.; Lange, A. W.; Lao, K. U.; Levine, D. S.; Liu, J.; McKenzie, S. C.; Morrison, A. F.; Nanda, K. D.; Plasser, F.; Rehn, D. R.; Vidal, M. L.; You, Z.-Q.; Zhu, Y.; Alam, B.; Albrecht, B. J.; Aldossary, A.; Alguire, E.; Andersen, J. H.; Athavale, V.; Barton, D.; Begam, K.; Behn, A.; Bellonzi, N.; Bernard, Y. A.; Berquist, E. J.; Burton, H. G. A.; Carreras, A.; Carter-Fenk, K.; Chakraborty, R.; Chien, A. D.; Closser, K. D.; Cofer-Shabica, V.; Dasgupta, S.; de Wergifosse, M.; Deng, J.; Diedenhofen, M.; Do, H.; Ehlert, S.; Fang, P.-T.; Fatehi, S.; Feng, Q.; Friedhoff, T.; Gayvert, J.; Ge, Q.; Gidofalvi, G.; Goldey, M.; Gomes, J.; González-Espinoza, C. E.; Gulania, S.; Gunina, A. O.; Hanson-Heine, M. W. D.; Harbach, P. H. P.; Hauser, A.; Herbst, M. F.; Hernández Vera, M.; Hodecker, M.;

Holden, Z. C.; Houck, S.; Huang, X.; Hui, K.; Huynh, B. C.; Ivanov, M.; Jász, Á.; Ji, H.; Jiang, H.; Kaduk, B.; Kähler, S.; Khistyaev, K.; Kim, J.; Kis, G.; Klunzinger, P.; Koczor-Benda, Z.; Koh, J. H.; Kosenkov, D.; Koulias, L.; Kowalczyk, T.; Krauter, C. M.; Kue, K.; Kunitsa, A.; Kus, T.; Ladjánszki, I.; Landau, A.; Lawler, K. V.; Lefrancois, D.; Lehtola, S.; Li, R. R.; Li, Y.-P.; Liang, J.; Liebenthal, M.; Lin, H.-H.; Lin, Y.-S.; Liu, F.; Liu, K.-Y.; Loipersberger, M.; Luenser, A.; Manjanath, A.; Manohar, P.; Mansoor, E.; Manzer, S. F.; Mao, S.-P.; Marenich, A. V.; Markovich, T.; Mason, S.; Maurer, S. A.; McLaughlin, P. F.; Menger, M. F. S. J.; Mewes, J.-M.; Mewes, S. A.; Morgante, P.; Mullinax, J. W.; Oosterbaan, K. J.; Paran, G.; Paul, A. C.; Paul, S. K.; Pavošević, F.; Pei, Z.; Prager, S.; Proynov, E. I.; Rák, Á.; Ramos-Cordoba, E.; Rana, B.; Rask, A. E.; Rettig, A.; Richard, R. M.; Rob, F.; Rossomme, E.; Scheele, T.; Scheurer, M.; Schneider, M.; Sergueev, N.; Sharada, S. M.; Skomorowski, W.; Small, D. W.; Stein, C. J.; Su, Y.-C.; Sundstrom, E. J.; Tao, Z.; Thirman, J.; Tornai, G. J.; Tsuchimochi, T.; Tubman, N. M.; Veccham, S. P.; Vydrov, O.; Wenzel, J.; Witte, J.; Yamada, A.; Yao, K.; Yeganeh, S.; Yost, S. R.; Zech, A.; Zhang, I. Y.; Zhang, X.; Zhang, Y.; Zuev, D.; Aspuru-Guzik, A.; Bell, A. T.; Besley, N. A.; Bravaya, K. B.; Brooks, B. R.; Casanova, D.; Chai, J.-D.; Coriani, S.; Cramer, C. J.; Cserey, G.; DePrince, A. E., III; DiStasio, R. A., Jr.; Dreuw, A.; Dunietz, B. D.; Furlani, T. R.; Goddard, W. A., III; Hammes-Schiffer, S.; Head-Gordon, T.; Hehre, W. J.; Hsu, C.-P.; Jagau, T.-C.; Jung, Y.; Klamt, A.; Kong, J.; Lambrecht, D. S.; Liang, W.; Mayhall, N. J.; McCurdy, C. W.; Neaton, J. B.; Ochsenfeld, C.; Parkhill, J. A.; Peverati, R.; Rassolov, V. A.; Shao, Y.; Slipchenko, L. V.; Stauch, T.; Steele, R. P.; Subotnik, J. E.; Thom, A. J. W.; Tkatchenko, A.; Truhlar, D. G.; Van Voorhis, T.; Wesolowski, T. A.; Whaley, K. B.; Woodcock, H. L., III; Zimmerman, P. M.; Faraji, S.; Gill, P. M. W.; Head-Gordon, M.; Herbert, J. M.; Krylov, A. I. Software for the Frontiers of Quantum Chemistry: An Overview of Developments in the Q-Chem 5 Package. *J. Chem. Phys.* **2021**, *155* (8), 084801. https://doi.org/10.1063/5.0055522.

(20) Liegeois Vincent. *DrawMol*, 2.1.20; UNamur: Namur, Belgium, 2024.

(21) *EasySpin - EPR spectrum simulation*. https://easyspin.org/ (accessed 2024-09-04).

(22) Stoll, S.; Schweiger, A. EasySpin, a Comprehensive Software Package for Spectral Simulation and Analysis in EPR. *J. Magn. Reson.* **2006**, *178* (1), 42–55. https://doi.org/10.1016/j.jmr.2005.08.013.
